# Effects of Different Dietary Supplements on Swimming Performance: A Systematic Review and Network Meta-Analysis

**DOI:** 10.3390/nu17010033

**Published:** 2024-12-26

**Authors:** Dongxiang Huang, Xiaobing Wang, Hideki Takagi, Shiwei Mo, Zhongzheng Wang, Daniel Hung-Kay Chow, Bo Huang

**Affiliations:** 1 School of Physical Education, Shaoguan University, Shaoguan 512005, China; hdx_ty@sgu.edu.cn (D.H.); wxb_ty@sgu.edu.cn (X.W.); 2 Department of Health and Physical Education, The Education University of Hong Kong, Hong Kong 999077, China; 3 Faculty of Health and Sport Sciences, University of Tsukuba, Tsukuba 3058555, Japan; takagi.hideki.ga@u.tsukuba.ac.jp; 4 School of Physical Education, Shenzhen University, Shenzhen 518060, China; moshiwei@szu.edu.cn; 5 School of Physical Education and Sports Science, South China Normal University, Guangzhou 510006, China; garywzz2@gmail.com

**Keywords:** swimmers, dietary supplements, swimming performance, network meta-analysis

## Abstract

Background: Nutritional supplements are widely used by swimmers, but the effectiveness of various supplements and the identification of the most effective intervention require further investigation. Purpose: This paper evaluated and compared the effectiveness of various nutrition-based interventions on swimming performance through both direct and indirect comparisons. Methods: PubMed, Embase, Web of Science, Cochrane Library, and SPORTDiscus databases were thoroughly searched up to 4 April 2024. The risk of bias was judged using the Cochrane risk of bias tool. A random-effect model was adopted to compute standardized mean differences (SMD) and 95% confidence intervals (CI). Results: L-arginine (Arg) demonstrated superior performance to the placebo (SMD = −1.66, 95% CI [−2.92, −0.44]), emerging as the most effective intervention for reducing 100 swimming time (SUCRA = 89.5%). Beta-alanine (BA) was the best intervention for improving blood lactate (SUCRA = 80%). Creatine combined with sodium bicarbonate (Creatine_NaHCO_3_) significantly increased blood pH compared to the placebo (SMD = 3.79, 95% CI [1.85, 5.80]), with a SUCRA score of 99.9%, suggesting it is the most effective intervention for this parameter. No prominent differences were noted among the interventions in 50 m time, 200 m time, heart rate, and body mass. Conclusions: Dietary supplements might provide benefits for improving swimming performance. Arg emerged as the most efficacious modality for reducing 100 m time. BA proved to be the preeminent strategy for decreasing blood lactate. Creatine_NaHCO_3_ was distinguished as the optimal approach for improving blood pH.

## 1. Introduction

Swimming represents an intensely competitive sport in which even minimal variations in time can significantly influence race results, particularly in short-distance competitions [1]. Consequently, athletes, coaches, and researchers are persistently exploring effective strategies to enhance swimming performance. Nutritional interventions have shown considerable promise in this pursuit [2].

Numerous clinical investigations have examined the effects of different dietary supplements, including caffeine [3], sodium bicarbonate (NaHCO_3_) [4], carbohydrates (CHO) [5], creatine [6], vitamin D (VD) [7], beta-alanine (BA) [8], beetroot [9], coenzyme Q10 (CoQ10) [10], royal jelly [11], vitamin E (VE) [12], L-carnitine (Carn) [13], L-arginine (Arg) [14], L-citrulline (Cit) [15], quercetin [16], probiotic [17], branched-chain amino acids (BCAAs) [18], rice germ (RG) [19], and chronic sodium citrate (CSC) [20], on swimming performance. However, only a few meta-analyses focus on nutritional supplementation strategies for swimming [21,22]. Meta-analyses on other supplements, such as CHO [5], creatine [6], VD [7], BA [8], beetroot [9], CoQ10 [10], royal jelly [11], VE [12], Carn [13], Arg [14], Cit [15], quercetin [16], probiotic [17], BCAAs [18], RG [19], and CSC [20], are lacking, partly due to an insufficient number of studies available for pairwise comparisons. Moreover, it is still uncertain which specific dietary supplement is the most effective. This uncertainty leads to inconclusive evidence from pairwise comparisons and fails to provide a robust basis for trainers, athletes, and practitioners to make informed decisions regarding swimming-related nutritional strategies. The effectiveness of various dietary supplements for swimmers and the most effective intervention necessitates additional investigation.

A network meta-analysis (NMA) can address these issues more comprehensively by incorporating both direct and indirect comparisons of multiple interventions [23]. Unlike pairwise meta-analyses, an NMA does not require experimental studies to have similar comparators. This feature significantly expands the evidence base and enables relative comparisons across various interventions [23]. Consequently, this approach allows for a comprehensive evaluation of all previously discussed swimming-specific findings within a single analysis and enables the ranking of interventions based on their effectiveness [24].

In this context, this study aimed to evaluate and compare the effectiveness of various nutrition-based interventions on variables related to swimming performance using both direct and indirect comparisons. The overall findings may aid athletes and coaches in selecting evidence-based strategies to enhance swimming performance and offer robust support for choosing optimal supplements in swimming.

## 2. Methods

This paper followed the PRISMA guidelines for NMA [25] and was pre-registered on PROSPERO (CRD42024548897).

### 2.1. Selection Criteria

Articles were deemed eligible if they met the following predetermined criteria: (1) participants: trained swimmers, irrespective of training level, age, or gender; (2) intervention: at least one of the dietary supplements (caffeine, CHO, NaHCO_3_, beetroot, creatine, BA, VD, CoQ10, royal jelly, VE, Carn, Arg, Cit, quercetin, probiotic, protein, BCAAs, and RG), within a reasonable range and with no limitation to any single supplement; (3) comparisons: the control group received a placebo intervention similar to the intervention group but without the active supplements; (4) outcomes: variables related to swimming performance, including the swimming time (e.g., 50 m time, 100 m time, and 200 m time), physiological metrics (e.g., blood lactate, heart rate, and blood pH), and body mass; and (5) study design: randomized control trials (RCTs) or randomized crossover designs (RCDs).

Conversely, studies were excluded if they met the following conditions: (1) participants were swimmers who had a history of medical ailments, diseases, or trauma; (2) the manuscript type was comments, editorials, or reviews; (3) there was no placebo group for result comparisons; (4) the full text was inaccessible, and direct contact with the authors for additional data was not possible; (5) the manuscript was not written in English and not published in a peer-reviewed journal.

Title, abstract, and full-text screening were undertaken independently by two investigators (D.H. and X.W.), and any disagreements were addressed through discussion with or adjudication by a third author (D.C.).

### 2.2. Search Strategy

PubMed, Web of Science, Embase, Cochrane Library, and SPORTDiscus were searched from the inception until 4 April 2024. Search records were retrieved using the following Boolean strategy without restrictions on region or year of publication: (“dietary supplement *” OR “food supplement *” OR “supplementation” OR “nutritional supplement *”) AND (swimming OR swimmer * OR swim). Table S1 lists the detailed search strategy.

### 2.3. Study Selection

Based on the predefined criteria, two researchers (D.H. and X.W.) independently conducted the study selection process. All potentially relevant articles were imported into EndNote X9 to eliminate duplicates. Titles and abstracts were then scanned to exclude ineligible studies. Finally, the full texts of potentially eligible articles were further evaluated, and those that met the inclusion criteria were used in the NMA. Any disagreements were addressed through discussion with a third researcher (D.C.).

### 2.4. Data Extraction

Pertinent data were extracted and entered into an Excel spreadsheet by two independent researchers (D.H. and X.W.). Any disagreements were addressed by a third author (B.H.). Study characteristics (e.g., first author, publication year, and geographic location), population features (e.g., sample size, gender, age, and training level), intervention characteristics (e.g., type, dose, and duration), and other relevant details (e.g., study design and outcomes) were extracted. Data graphically recorded as means and standard deviations (SDs) were extracted using Web Plot Digitizer V4.0 (Free Software Foundation, Boston, MA, USA) [26]. The reported standard errors were converted to SDs to ensure consistency. In RCTs with multiple time points, only the final measurement was included. For missing information, the corresponding author was contacted to provide the necessary information for analyses.

### 2.5. Risk of Bias

The risk of bias was judged through the Cochrane risk of bias tool for RCTs [27] by two investigators (D.H. and X.W.) independently across seven domains: random sequence generation, allocation concealment, the blinding of participants and personnel, the blinding of outcome assessment, incomplete outcome data, selective reporting, and other biases. Each domain was graded as low, high, or unclear risk of bias. Any disagreements were resolved through discussion with a third author (B.H.).

### 2.6. Publication Bias

To explore the potential publication bias in the combined data from each study, a visual assessment of the funnel plot’s asymmetry was performed. This method facilitated the evaluation of bias in the dissemination of research included in the review.

### 2.7. Statistical Analysis

Given the different scoring scales and measurement methods across the included studies, standardized mean differences (SMD) with a 95% confidence interval (CI) were employed as the effect size to enable comparisons across studies measuring the same outcome on different scales. This approach ensures normalized outcomes, facilitating consistent and reliable data integration in the NMA.

To ensure robust analysis, a random-effect model based on the Bayesian Markov chain Monte Carlo method was utilized to acquire non-informative prior distributions [28]. This model accounted for heterogeneity across populations, interventions, and methodologies. The Bayesian approach was particularly well suited for this study, as it integrates both direct and indirect evidence to estimate relative intervention effects while generating robust posterior distributions for parameter estimation, thereby enhancing result reliability.

Global inconsistency was assessed by computing the deviance information criterion (DIC) for both consistency and inconsistency models. The difference in DIC (dDIC) evaluated the agreement between direct and indirect evidence, with dDIC < 10 indicating no significant global inconsistency [29]. To further ensure model validity, local inconsistency was examined using a node-split model, which identifies discrepancies between direct and indirect evidence for specific comparisons. A *p*-value < 0.05 in the node-split analysis indicates notable local inconsistency, warranting further investigation or model adjustments [30]. Together, these methods ensure that the conclusions drawn from the NMA are based on robust and consistent evidence.

The surface under the cumulative ranking curve (SUCRA), derived from posterior probabilities, ranked the relative effectiveness of various interventions [31]. SUCRA quantifies an intervention’s relative ranking probability, with higher values indicating a higher likelihood of ranking among the most effective options. This ranking simplifies comparisons and offers practical insights into the relative effectiveness of dietary supplements. Stata 15.0 (Stata Corporation, College Station, TX, USA) and R 4.4.1 (R Development Core Team, Vienna, http://www.R-project.org accessed on 16 June 2024) were applied for the NMA.

## 3. Results

### 3.1. Literature Selection

**Figure 1:**
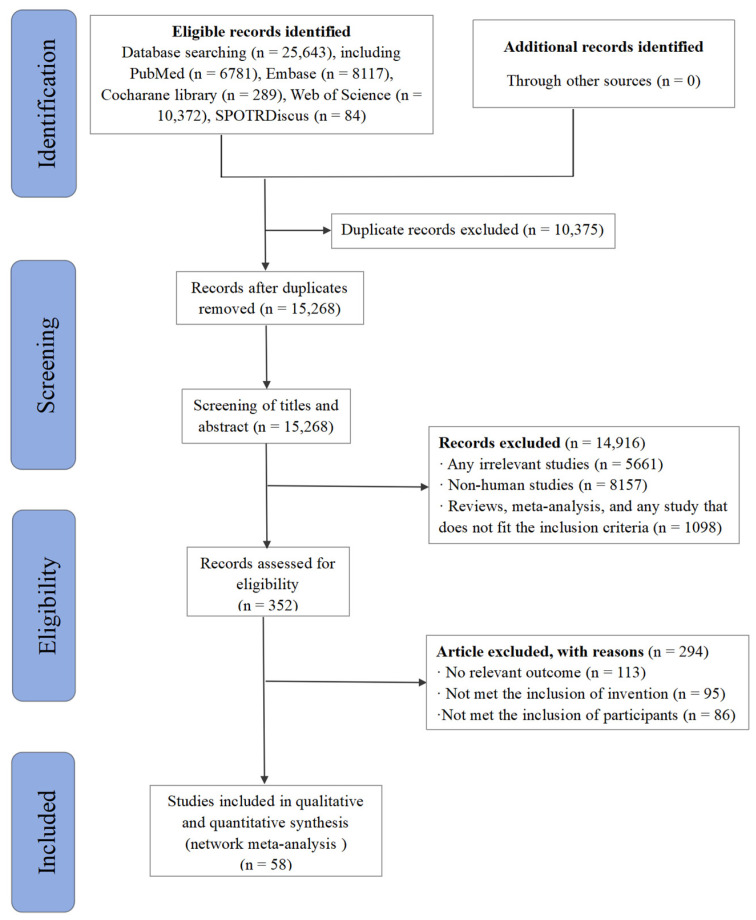
PRISMA flow diagram of article selection.

From the initial pool of 25,643 publications identified through our literature search, 15,268 records remained after the removal of duplicates. Upon screening the titles and abstracts, 352 articles were selected for further assessment. Subsequently, 294 studies were excluded due to irrelevant outcomes (*n* = 113) and not meeting the inclusion criteria regarding intervention (*n* = 95) and participants (*n* = 86). Ultimately, 58 studies [3,4,5,6,7,8,9,10,11,12,13,14,15,16,17,18,19,20,32,33,34,35,36,37,38,39,40,41,42,43,44,45,46,47,48,49,50,51,52,53,54,55,56,57,58,59,60,61,62,63,64,65,66,67,68,69,70,71] were incorporated into this NMA (Figure 1).

### 3.2. Characteristics of Studies

In total, 58 articles involving 1014 participants were published between 1971 and 2024, spanning 18 countries, with most in the United Kingdom (*n* = 11). Most studies were RCTs (*n* = 44), and the rest were RCDs (*n* = 14), with sample sizes of 6–47 participants. The studies investigated 18 different dietary supplements, including creatine (*n* = 17), NaHCO_3_, (*n* = 9), caffeine (*n* = 5), beetroot (*n* = 4), CHO (*n* = 4), BA (*n* = 4), Arg (*n* = 3), VD (*n* = 2), CoQ10 (*n* = 1), royal jelly (*n* = 1), VE (*n* = 1), Carn (*n* = 1), Cit (*n* = 1), quercetin (*n* = 1), probiotics (*n* = 1), protein (*n* = 1), BCAAs (*n* = 1) and RG (*n* = 1). Other combinations of supplements, each represented by a single study, included Arg combined with Cit and BCAAs (Arg_Cit_BCAAs), BA combined with NaHCO_3_ (BA_NaHCO_3_), NaHCO_3_ combined with caffeine (NaHCO_3__Caffeine), and creatine combined with NaHCO_3_ (Creatine_NaHCO_3_).

The dose ranges for the investigated supplements varied significantly across studies. For creatine, doses ranged from 2 to 25 g/day, while NaHCO_3_ was administered at doses from 2.9 mmol/kg to 0.31 g/kg of body mass, and caffeine ranged from 3 to 250 mg/kg of body mass. Other supplements showed similar variability: beetroot ranged from 70 mL/day to 0.5 L/day, CHO from 0.5 to 12 g/kg of body mass, BA from 2 to 6.4 g/day, Arg from 7 to 8 g/day, and VD from 2000 to 5000 IU/day. For supplements reported in single studies, the doses included CoQ10 (300 mg/day), royal jelly (2 g/day), VE (400 mg/day), Carn (4 g/day), Cit (8 g/day), quercetin (1000 mg/day), probiotics (4 mg/day), protein (0.5 g/kg of body mass), BCAAs (0.085 g/kg of body mass), and RG (25 g/day). Several combined interventions were also investigated. For Arg_Cit_BCAAs, BCAAs were administered at a dose of 0.085 g/kg of body mass, combined with Cit and Arg at 0.05 g/kg of body mass each. BA_NaHCO_3_ consisted of 4.8 g/day BA combined with 0.3 g/kg of body mass NaHCO_3_, while NaHCO_3__Caffeine included 6.2 ± 0.3 mg/kg of body mass caffeine combined with 0.3 g/kg of body mass NaHCO_3_. Lastly, Creatine_NaHCO_3_ was administered as 20 g/day creatine combined with 0.3 g/kg of body mass NaHCO_3_. Intervention durations varied from 2 to 84 days. The detailed characteristics of the articles are displayed in Table S2.

### 3.3. Swimming Time

#### 3.3.1. The 50 m Time

**Figure 2:**
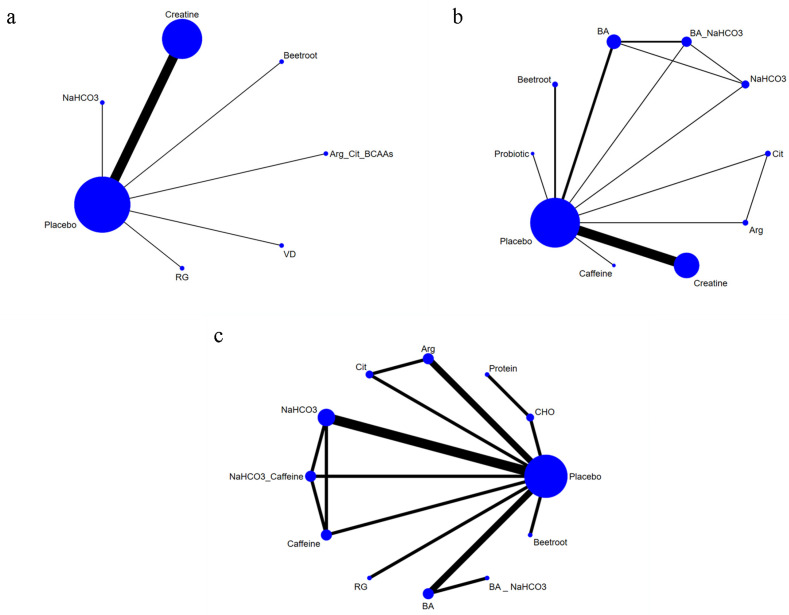
Network of intervention comparisons: (**a**) 50 m time; (**b**) 100 m time; (**c**) 200 m time.

We performed a comprehensive NMA encompassing 17 studies and 358 participants to evaluate the effects of various dietary supplements on the 50 m time. The network plots included seven distinct nodes representing various dietary supplements and six direct comparisons (Figure 2a); creatine was the most investigated intervention.

**Figure 3:**
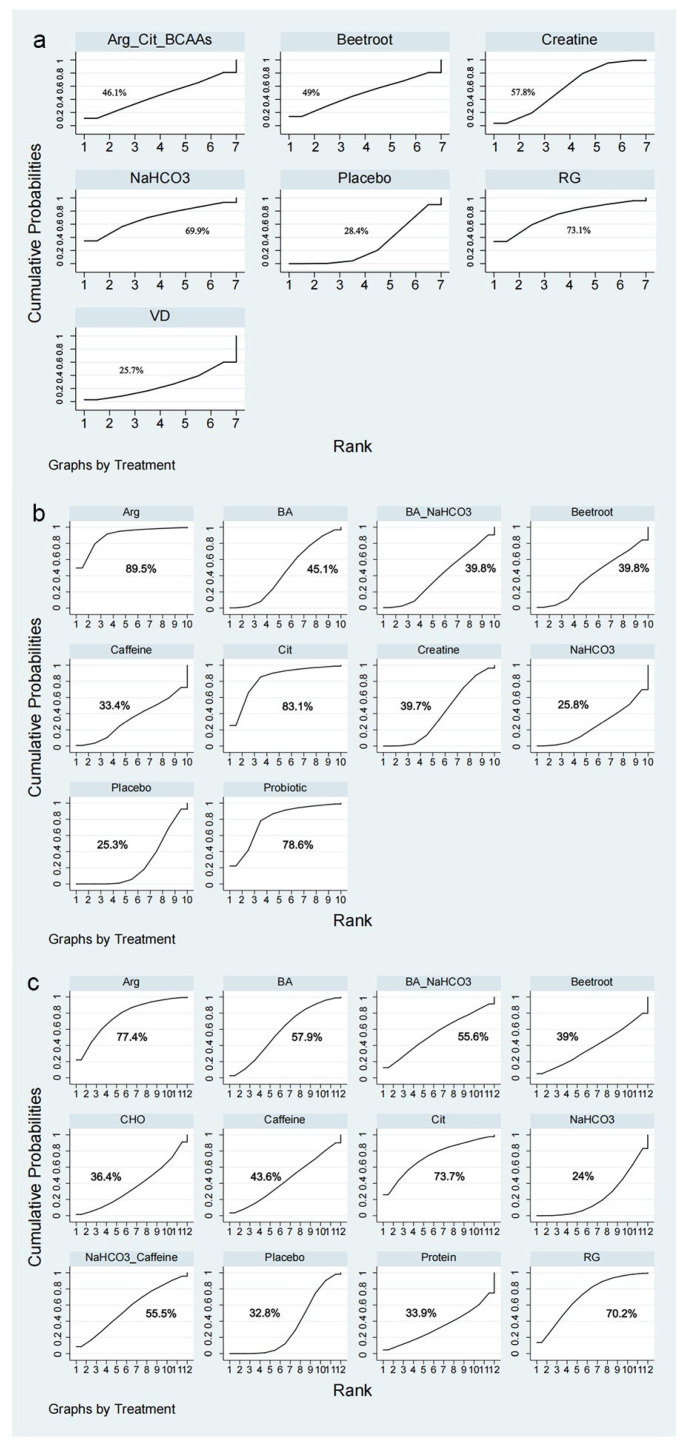
Ranking of nutritional supplement interventions based on the probability of their effects: (**a**) 50 m time; (**b**) 100 m time; (**c**) 200 m time.

According to the SUCRA, RG may be the most effective dietary supplement for decreasing the 50 m time (SUCRA = 73.1%) (Figure 3a). However, pairwise comparisons revealed no significant differences in the 50 m time among the different dietary supplements (Table 1).

#### 3.3.2. The 100 m Time

Notably, 19 studies, encompassing 300 participants, recorded the 100 m time. The network plots featured 10 distinct nodes representing various dietary supplements and 13 direct comparisons (Figure 2b). Among these studies, BA was the most frequently evaluated supplement.

The SUCRA probability analysis evinced that the most effective dietary supplement for reducing the 100 m time might be Arg with a SUCRA score of 89.5%, followed by Cit (SUCRA = 83.1%) (Figure 3b). Pairwise comparisons unveiled that Arg demonstrated enhanced effectiveness than BA (SMD = −1.51, 95% CI [−2.88, −0.18]), BA_NaHCO_3_ (SMD = −1.56, 95% CI [−2.98, −0.18]), creatine (SMD = −1.52, 95% CI [−2.81, −0.25]), NaHCO_3_ (SMD = −1.79, 95% CI [−3.23, −0.36]), and placebo (SMD = −1.66, 95% CI [−2.92, −0.44]). Similarly, Cit was significantly more effective than NaHCO_3_ (SMD = −1.54, 95% CI [−2.93, −0.09]) and the placebo (SMD = −1.41, 95% CI [−2.63, −0.15]) (Table 2).

#### 3.3.3. The 200 m Time

The 200 m time was evaluated in 10 studies involving 132 participants. The network plots comprised 12 distinct nodes representing various dietary supplements and 15 direct comparisons (Figure 2). NaHCO_3_ was the most frequently evaluated supplement.

Based on the SUCRA, Arg (SUCRA = 77.4%) may be the most effective intervention for diminishing the 200 m time (Figure 2c). However, no significant differences in the 200 m time were noted among the dietary supplements (Table 3).

### 3.4. Physiological Indicators

#### 3.4.1. Blood Lactate

Blood lactate was assessed in 31 studies involving 528 participants. The network diagram included 17 distinct interventions and 23 direct comparisons (Figure S1). NaHCO_3_ and caffeine were the most evaluated dietary supplements.

The SUCRA probability analysis suggested that BA might be the most effective intervention for reducing blood lactate (SUCRA = 80%), closely followed by beetroot (SUCRA = 77.3%) (Figure S2). Pairwise comparisons demonstrated that BA had greater efficacy than Creatine_NaHCO_3_ (SMD = −2.66, 95% CI [−4.29, −0.98]), NaHCO_3_ (SMD = −0.84, 95% CI [−1.52, −0.16]), and NaHCO_3__Caffeine (SMD = −1.85, 95% CI [−2.86, −0.84]). Similarly, beetroot outperformed Creatine_NaHCO_3_ (SMD = −2.72, 95% CI [−4.46, −0.98]) and NaHCO_3__Caffeine (SMD = −1.91, 95% CI [−3.12, −0.75]). Detailed results of other pairwise comparisons are summarized in Table S3.1.

#### 3.4.2. Blood pH

In total, 12 studies encompassing 189 participants investigated Blood pH. The network diagram (Figure S1) included 9 distinct interventions and 14 direct comparisons. NaHCO_3_ was identified as the most evaluated dietary supplement.

Based on the SUCRA, Creatine_NaHCO_3_ (SUCRA = 99.9%) appeared to be the most effective intervention for increasing blood pH, followed by BA_NaHCO_3_ (SUCRA = 77.8%) (Figure S2). Additionally, pairwise comparison indicated that compared to the placebo, BA_NaHCO_3_ (SMD = 1.09, 95% CI [0.32, 1.85]), Creatine_NaHCO_3_ (SMD = 3.79, 95% CI [1.85, 5.80]), and NaHCO_3_ (SMD = 0.93, 95% CI [0.49, 1.39]) significantly increased blood pH. The comprehensive outcomes of additional pairwise comparisons are presented in Table S3.2.

#### 3.4.3. Heart Rate

Heart rate was estimated in 13 studies encompassing 226 participants. The network diagram (Figure S1) included eight distinct interventions and seven direct comparisons. Creatine and beetroot were the most compared dietary supplements.

According to the SUCRA, beetroot (SUCRA = 72.1%) may be the most effective intervention for reducing the heart rate (Figure S2). However, the dietary supplements did not result in significant differences in the heart rate (Table S3.3).

### 3.5. Body Mass

Body mass was examined in 12 studies involving 244 participants. The network diagram (Figure S1) encompassed six distinct interventions and five direct comparisons. Creatine was identified as the most frequently evaluated dietary supplement.

The SUCRA probability analysis exhibited that VD was the most effective intervention for reducing body mass with a SUCRA score of 63.2% (Figure S2). However, no marked differences in weight loss were observed across interventions (Table S3.4).

### 3.6. Risk of Bias

All eligible studies reported randomization and were categorized as “low risk” of bias. In terms of allocation concealment, 30 articles were rated as “unclear risk”, and one article was identified as “high risk”. Regarding the blinding of participants and personnel, 13 articles were assessed as “unclear risk,” while 4 articles were deemed “high risk”. For the blinding of outcome assessment, 56 articles exhibited an “unclear risk”. Notably, 8 articles were considered “high risk” concerning complete data, whereas 58 articles demonstrated no evidence of selective outcome reporting or other biases (Figure S3 and Table S4).

### 3.7. Publication Bias

The funnel plot (Figure S4) showed that all studies were symmetrically distributed on either side of the vertical line at X = 0, suggesting the absence of significant publication bias.

### 3.8. Exploration of Inconsistency

All outcomes demonstrated satisfactory model fitting and iteration convergence. The results indicated no notable difference between the global consistency model and the inconsistency model (Table S5). Several closed-loop network configurations were identified when comparing the 100 m time, blood lactate, and blood pH. The node-splitting results revealed no inconsistencies between direct and indirect evidence (all *p* > 0.05, Table S6).

## 4. Discussion

This systematic review and NMA first investigated the effects of various dietary supplements on swimming performance, and the key findings supported the benefits of dietary supplements. The analysis confirmed that specific dietary supplements can significantly enhance swimming performance outcomes. Notably, (1) Arg outperformed the placebo and other interventions, potentially being the most effective in reducing the 100 m time, followed by Cit; (2) BA was the most effective in reducing blood lactate; (3) compared to the placebo, BA_NaHCO_3_, Creatine_NaHCO_3_, and NaHCO_3_ significantly increased blood pH, with Creatine_NaHCO_3_ being possibly the best intervention for increasing blood pH; (4) no significant differences were observed in the 50 m time, 200 m time, heart rate, and body mass among interventions.

This NMA discovered that both Arg and Cit significantly reduced the 100 m time, exhibiting high SUCRA values. These amino acids enhance athletic performance by increasing power output and prolonging endurance, largely due to the increased production of nitric oxide (NO) [72]. NO is a critical physiological signaling molecule that enhances blood flow to skeletal muscle, optimizes metabolic processes, boosts contractile function, and mitigates fatigue [72]. It enhances exercise performance by promoting exercise-induced vasodilation, muscle oxygenation, and VO_2_ kinetics [73]. Arg and Cit supplementation can enhance swimming durations during high-intensity interval training among young swimmers [18].

Our NMA results indicated that Arg is the most effective supplement for reducing 100 m swimming time, outperforming both the placebo and other interventions, with Cit following closely behind. This finding reflects the cumulative evidence across multiple studies. However, inconsistencies regarding the efficacy, dosage, and supplementation duration become apparent when examining individual studies included in this analysis. For Arg, evidence suggests that a multi-dose regimen of 7 g/day, divided into three doses (morning, afternoon, and evening) for 7 days, provides the most potential benefits, particularly in improving hormone balance (e.g., increased growth hormone and testosterone, decreased cortisol, etc.) [14]. However, this regimen did not directly enhance short-duration, high-intensity swimming performance. In contrast, a single or twice-daily dose of 8 g/day for 7–8 days showed no significant effects on performance or blood lactate levels [15,40]. These findings suggest that multi-dose administration may be more suitable for recovery purposes, while the impact of higher doses (e.g., >10 g/day) and longer supplementation durations (>10 days) on performance requires further investigation. For Cit, limited evidence is available. One study [15] using 8 g/day for 8 days reported no significant improvements in swimming performance or plasma NOx levels. Although Cit theoretically benefits performance by promoting nitric oxide production and enhancing blood flow, current dosing and timing strategies appear insufficient to fully exploit its potential. Future research should focus on exploring higher doses (e.g., >10 g/day) and extended supplementation periods (>14 days) to evaluate their effects on high-intensity performance.

The discrepancies between our NMA results and individual studies highlight the critical role of dosage and supplementation timing. By integrating data from multiple studies, the NMA may capture subtle cumulative effects that individual trials might not reveal, particularly under varying dosage and timing conditions [74]. Additionally, differences in study design—including dosage, duration, the timing of administration, and participants’ training status—can significantly influence outcomes. For instance, while two studies [15,40] found no significant effects from short-term (7–8 days), high-dose (8 g/day) Arg supplementation, Moosakhani et al. (2018) demonstrated that a multi-dose regimen positively affected hormone balance [14]. These findings underscore the need for future research to (1) compare multi-dose versus single large-dose supplementation, particularly at different time points (e.g., pre-competition vs. recovery period); (2) explore the impact of higher Arg doses (e.g., >10 g/day) and longer supplementation periods (>10 days) on performance and recovery; and (3) investigate higher doses and longer supplementation periods for Cit (e.g., >10 g/day for >14 days) alongside biomarkers (e.g., plasma NOx levels) to better understand the underlying mechanisms.

Although Arg and Cit supplementation notably influenced the 100 m time, our NMA indicated that other dietary supplements, such as caffeine and NaHCO_3_, had no significant effect compared to the placebo. A meta-analysis demonstrated that caffeine significantly reduced swimming time [21], which was opposite to our findings. This inconsistency may stem from different inclusion criteria. Our study conducted subgroup analyses on 100 m and 200 m, while Grgic’s study combined various distances (75 m, 100 m, and 200 m), potentially introducing bias. Another meta-analysis consistently indicated that NaHCO_3_ did not affect the 100 m swimming time [22]. Among all the interventions, no significant differences were observed in the 50 m and 200 m swimming times. This lack of effect may be attributed to the distinct metabolic demands of these events. The 50 m event relies primarily on intramuscular phosphocreatine and anaerobic glycolysis [64], which may limit the impact of supplements like Arg and Cit that enhance nitric oxide (NO)-mediated vasodilation and oxygen delivery [72]. Conversely, the 200 m event involves a hybrid energy system combining aerobic and anaerobic metabolism [75], potentially diluting the ergogenic effects of certain supplements. Additionally, the absence of significant findings may reflect variations in study design, such as small sample sizes, differences in supplementation protocols, and individual variability in supplement responses.

Regarding physiological indicators, SUCRA analysis demonstrated that BA was the most effective intervention for reducing blood lactate. This effect is primarily attributed to the role of carnosine, a dipeptide formed by BA and histidine, which functions as an intracellular buffer during high-intensity exercise. Long-term BA supplementation can significantly increase muscle carnosine levels, enhancing buffering capacity, circulatory efficiency, and ventilatory thresholds, thereby delaying fatigue during exercise [76,77,78]. During high-intensity anaerobic exercise, lactate is produced via anaerobic metabolic pathways [79]. The dissociation of lactate into lactate salts and hydrogen ions (H+) in the cellular environment leads to acidosis and fatigue [52]. Carnosine neutralizes H+ accumulation, maintaining acid–base balance within cells [80]. Furthermore, carnosine’s antioxidant properties mitigate oxidative stress by neutralizing reactive oxygen species (ROS) produced during muscle contraction, further enhancing exercise performance [81]. These combined effects explain why BA supplementation, through increased carnosine levels, emerges as the most effective intervention for reducing blood lactate. However, given that only two trials have specifically examined BA’s impact on swimmers’ blood lactate, these findings should be interpreted cautiously.

Building upon these physiological insights, current evidence regarding the optimal dosing and timing of BA supplementation highlights a protocol of 4–6 g/day over 2–5 weeks as the most effective for improving short-duration, high-intensity exercise performance. This regimen has been shown to significantly delay fatigue by enhancing buffering capacity, particularly during high-intensity efforts. Three studies support a staged supplementation approach, where BA is administered at 2–3 g/day initially, gradually increasing to 4–6 g/day over 14 days, resulting in an improved first ventilatory threshold (VT1) but limited effects on the second ventilatory threshold (VT2) [8]. Comparatively, the continuous supplementation of 6.4 g/day (divided into four doses) for 5 weeks improved swimming performance by 2.0–2.1% [38]. Conversely, Mero et al. (2013) reported that a dosage of 4.8 g/day produced only short-term benefits, with effects diminishing after 4 weeks [52]. These findings suggest that 4–6 g/day over 2–5 weeks is an optimal protocol, while lower doses or prolonged supplementation may result in limited benefits due to carnosine saturation.

High-intensity exercise results in significant lactate accumulation, leading to the dissociation of lactate into lactate ions and hydrogen ions (H+), which decreases intracellular and extracellular pH. This drop in pH causes metabolic acidosis, impairing muscle contractility and energy production, and ultimately inducing fatigue [63,64]. Our NMA results demonstrated that BA_NaHCO_3_, Creatine_NaHCO_3_, and NaHCO_3_ effectively increased blood pH compared to the placebo. Among these, Creatine_NaHCO_3_ appeared to be the most effective intervention for enhancing blood pH. By maintaining a higher blood pH, metabolic alkalosis counteracts the rise in H+ concentration [8], thereby delaying fatigue onset and sustaining muscle contractile function during repeated high-intensity efforts. The combined supplementation of creatine and NaHCO_3_ exerts ergogenic effects through complementary mechanisms. Creatine enhances intracellular phosphocreatine (PCr) stores, supporting rapid ATP resynthesis during short-duration, high-intensity exercise [43]. Meanwhile, NaHCO_3_ acts extracellularly to improve blood buffering capacity, mitigating the effects of H+ accumulation during intense anaerobic activity [48]. These synergistic actions provide a dual mechanism to combat fatigue, making this combination particularly effective for intermittent, high-intensity performance, such as repeated sprint swimming [51].

Regarding dosing and timing, evidence from Mero et al. (2004) suggests an optimal protocol for Creatine_NaHCO_3_ supplementation [51]. The study reported that 20 g/day of creatine (divided into four doses) over six consecutive days, combined with 0.3 g/kg body weight of sodium bicarbonate ingested two hours prior to testing, significantly improved the second 100 m sprint performance by approximately 0.9 s. However, as this conclusion is based on a single study, the reliability and generalizability of this dosing protocol remain uncertain. Further high-quality randomized controlled trials are warranted to confirm these findings, refine supplementation strategies, and validate their efficacy across diverse athletic contexts and populations.

## 5. Strengths and Limitations

This NMA exclusively includes trained swimmers, thereby reducing clinical heterogeneity and enhancing result comparability. Common dietary supplements in adjuvant therapy were thoroughly searched, incorporating enough RCTs and comparing multiple nutrients. The stability and reliability of the NMA hinge on uniform standards of similarity, homogeneity, and consistency. No inconsistencies were detected, confirming the robustness of the results. SUCRA and cluster rank analyses confirm that the obtained findings are valuable for clinical decision-making.

Nevertheless, several limitations of this NMA should be acknowledged. First, the small sample sizes and limited studies may impact the accuracy and generalizability of our findings. Second, heterogeneity may arise due to different patient treatment protocols and different formulations, dosages, administration routes, and the timing of nutritional supplements across studies. Third, this NMA was restricted to research published in English, which may result in selection bias. Therefore, additional high-quality RCTs are necessary to validate our conclusions.

This study specifically included RCTs or RCDs evaluating dietary supplement interventions to ensure methodological consistency and rigor. As a result, observational studies, such as Lukaski et al. (1996) [82], which investigated the effects of iron, copper, magnesium, and zinc status on swimming performance, were excluded. While these studies provide valuable insights into dietary and physiological factors influencing swimming performance, they fall outside the methodological scope of our analysis. Future research should focus on well-designed RCTs to provide stronger evidence regarding the effects of mineral status on swimming performance.

The study’s scope also presents specific limitations. The analysis was confined to three swimming distances (50 m, 100 m, and 200 m), which may limit its applicability to other race distances. Expanding future analyses to include longer distances, such as 400 m and 1500 m, could improve the relevance and comprehensiveness of the findings across different competitive contexts.

Furthermore, this study primarily focused on the short-term effects of dietary supplementation on swimming performance, restricting the generalizability of the findings to long-term scenarios. Long-term supplementation might yield different outcomes for both performance and health. Future research should evaluate the long-term impacts of dietary supplements to provide a deeper understanding of their sustained benefits and risks.

Lastly, although combination supplements such as Arg_Cit_BCAAs, BA_NaHCO_3_, NaHCO_3__Caffeine, and Creatine_NaHCO_3_ were assessed, data on potential negative interactions between these supplements remain limited. This constraint hampers a comprehensive evaluation of their safety profiles. Future research should focus on investigating the safety and potential adverse interactions of combination supplements to enable more informed and practical applications.

## 6. Conclusions

Nutritional supplements could be beneficial interventions for enhancing swimming performance. Arg and Cit seemed to be the most effective for improving the 100 m time. BA and Creatine_NaHCO_3_ showed superior effects on blood lactate and blood pH, respectively. Additional investigation is warranted to validate these findings.

## Figures and Tables

**Table 1:** League table of swimming performance for the 50 m time.

Arg_Cit_BCAAs	-	-	-	-	-	-
0.07 (−2.44, 2.56)	Beetroot	-	-	-	-	-
0.16 (−1.62, 2.01)	0.08 (−1.74, 1.98)	Creatine	-	-	-	-
0.54 (−1.98, 3.07)	0.47 (−2.08, 3.02)	0.39 (−1.53, 2.23)	NaHCO_3_	-	-	-
−0.2 (−1.96, 1.53)	−0.28 (−2.08, 1.52)	−0.36 (−0.91, 0.1)	−0.75 (−2.57, 1.06)	Placebo	-	-
0.6 (−1.81, 2.99)	0.53 (−1.9, 2.97)	0.45 (−1.31, 2.12)	0.06 (−2.4, 2.51)	0.81 (−0.84, 2.45)	RG	-
−0.4 (−2.75, 1.95)	−0.47 (−2.85, 1.92)	−0.55 (−2.24, 1.06)	−0.94 (−3.34, 1.46)	−0.19 (−1.76, 1.38)	−1 (−3.25, 1.29)	VD

**Table 2:** League table of swimming performance for 100 m time.

Arg	-	-	-	-	-	-	-	-	-
−1.51 (−2.88, −0.18)	BA	-	-	-	-	-	-	-	-
−1.56 (−2.98, −0.18)	−0.05 (−0.66, 0.55)	BA_NaHCO_3_	-	-	-	-	-	-	-
−1.52 (−3.03, 0.01)	0 (−1.06, 1.07)	0.05 (−1.07, 1.19)	Beetroot	-	-	-	-	-	-
−1.61 (−3.29, 0.05)	−0.08 (−1.36, 1.17)	−0.04 (−1.36, 1.28)	−0.09 (−1.56, 1.37)	Caffeine	-	-	-	-	-
−0.26 (−1.43, 0.88)	1.26 (−0.13, 2.6)	1.32 (−0.11, 2.7)	1.27 (−0.3, 2.77)	1.34 (−0.33, 2.99)	Cit	-	-	-	-
−1.52 (−2.81, −0.25)	−0.01 (−0.65, 0.66)	0.04 (−0.69, 0.81)	−0.01 (−0.98, 0.98)	0.08 (−1.1, 1.3)	−1.27 (−2.52, 0.05)	Creatine	-	-	-
−1.79 (−3.23, −0.36)	−0.27 (−0.97, 0.42)	−0.22 (−0.94, 0.5)	−0.27 (−1.44, 0.9)	−0.18 (−1.52, 1.18)	−1.54 (−2.93, −0.09)	−0.26 (−1.08, 0.52)	NaHCO_3_	-	-
−1.66 (−2.92, −0.44)	−0.16 (−0.7, 0.39)	−0.1 (−0.76, 0.56)	−0.15 (−1.07, 0.75)	−0.07 (−1.2, 1.09)	−1.41 (−2.63, −0.15)	−0.14 (−0.52, 0.2)	0.12 (−0.6, 0.83)	Placebo	-
−0.56 (−2.3, 1.14)	0.94 (−0.37, 2.28)	1 (−0.37, 2.38)	0.95 (−0.57, 2.44)	1.04 (−0.61, 2.69)	−0.31 (−2.02, 1.43)	0.95 (−0.31, 2.21)	1.21 (−0.19, 2.63)	1.1 (−0.1, 2.31)	Probiotic

**Table 3:** League table of swimming performance for 200 m time.

Arg	-	-	-	-	-	-	-	-	-	-	-
−0.53 (−1.81, 0.75)	BA	-	-	-	-	-	-	-	-	-	-
−0.54 (−2.33, 1.29)	0 (−1.24, 1.24)	BA_NaHCO_3_	-	-	-	-	-	-	-	-	-
−0.88 (−2.56, 0.85)	−0.34 (−1.97, 1.33)	−0.33 (−2.41, 1.75)	Beetroot	-	-	-	-	-	-	-	-
−0.7 (−1.98, 0.59)	−0.17 (−1.4, 1.06)	−0.17 (−1.92, 1.58)	0.18 (−1.52, 1.8)	Caffeine	-	-	-	-	-	-	-
−0.91 (−2.49, 0.67)	−0.38 (−1.91, 1.16)	−0.38 (−2.35, 1.6)	−0.04 (−1.95, 1.85)	−0.22 (−1.74, 1.31)	CHO	-	-	-	-	-	-
−0.08 (−1.2, 1.04)	0.46 (−0.98, 1.87)	0.46 (−1.46, 2.34)	0.8 (−1.02, 2.58)	0.62 (−0.81, 2.05)	0.82 (−0.86, 2.54)	Cit	-	-	-	-	-
−0.92 (−2.1, 0.24)	−0.39 (−1.46, 0.68)	−0.38 (−2.03, 1.25)	−0.04 (−1.62, 1.51)	−0.22 (−1.09, 0.65)	0 (−1.43, 1.41)	−0.84 (−2.14, 0.46)	NaHCO_3_	-	-	-	-
−0.47 (−1.76, 0.8)	0.06 (−1.15, 1.28)	0.07 (−1.68, 1.8)	0.41 (−1.29, 2.07)	0.23 (−0.69, 1.17)	0.45 (−1.09, 1.96)	−0.39 (−1.81, 1.04)	0.44 (−0.41, 1.33)	NaHCO_3__Caffeine	-	-	-
−0.9 (−1.85, 0.05)	−0.36 (−1.23, 0.5)	−0.36 (−1.89, 1.15)	−0.02 (−1.47, 1.37)	−0.2 (−1.06, 0.68)	0.02 (−1.25, 1.27)	−0.82 (−1.95, 0.32)	0.02 (−0.63, 0.69)	−0.43 (−1.29, 0.44)	Placebo	-	-
−1 (−2.91, 0.93)	−0.46 (−2.32, 1.42)	−0.46 (−2.7, 1.78)	−0.12 (−2.31, 2.06)	−0.29 (−2.15, 1.57)	−0.08 (−1.18, 1.02)	−0.91 (−2.94, 1.09)	−0.08 (−1.84, 1.71)	−0.52 (−2.39, 1.34)	−0.09 (−1.76, 1.57)	Protein	-
−0.32 (−1.7, 1.07)	0.21 (−1.11, 1.56)	0.22 (−1.62, 2.05)	0.56 (−1.22, 2.29)	0.38 (−0.97, 1.74)	0.59 (−1.01, 2.25)	−0.24 (−1.77, 1.31)	0.6 (−0.61, 1.82)	0.15 (−1.18, 1.51)	0.58 (−0.44, 1.6)	0.68 (−1.28, 2.61)	RG

## Data Availability

Data can be obtained from the corresponding author upon reasonable request due to the need for clarification or additional details regarding the data extraction and analysis process performed for this network meta-analysis. All data supporting this study are publicly available in the original literature, and there are no privacy, legal, or ethical restrictions associated with the data.

## Supplementary Materials

The following supporting information can be downloaded at: https://www.mdpi.com/article/10.3390/nu17010033/s1, Table S1: Search strategy; Table S2: Characteristics of studies included in the review; Table S3.1: League table of swimming performance for blood lactate; Table S3.2: League table of swimming performance for blood pH; Table S3.3: League table of swimming performance for heart rate; Table S3.4: League table of swimming performance for body mass; Table S4: Overall risk of bias assessment for each study; Table S5: Global inconsistency and heterogeneity of each outcome; Table S6. Node-splitting results; Figure S1: Network of intervention comparisons. a: blood lactate, b: blood pH, c: heart rate, d: body mass; Figure S2: Ranking of nutritional supplement interventions based on the probability of their effects. a: blood lactate, b: blood pH, c: heart rate, d: body mass; Figure S3: Risk of bias; Figure S4: Publication bias for a: 50 m time, b:100m time, c: 200 m time, d: blood lactate, e: blood pH, f: heart rate, g: body mass.
