# Supplementary material for: Effects of Different Dietary Supplements on Swimming Performance: A Systematic Review and Network Meta-Analysis
Source: Nutrients. 2024 Dec 26;17(1):33. doi: 10.3390/nu17010033 (PMC11722695; doi:10.3390/nu17010033)
Supplement: Supplementary file 1 [file nutrients-17-00033-s001.zip › nutrients-3383375-supplementary.pdf]

Supplementary materials

Table S1. Search Strategy 2

Table S2. Characteristics of studies included in the review 4

Figure S1. Network of intervention comparisons. a: blood lactate, b: blood pH, c: heart rate, d: body mass 10

Table S3. League table of swimming performance 12

Figure S2. Ranking of nutritional supplement interventions based on the probability of their effects. a: blood lactate, b: blood pH, c: heart rate, d: body mass 17

Figure S3. Risk of bias 19

Table S4. Overall risk of bias assessment for each study 20

Figure S4. Publication bias for a: 50 m time, b:100m time, c: 200 m time, d: blood lactate, e: blood pH, f: heart rate, g: body mass 23

Table S5. Global inconsistency and heterogeneity of each outcome 27

Table S6. Node-splitting results 28

Table S1. Search strategy

Databases	Search number	Query	Results
Embase	#1	'dietary supplements':ti,ab,kw OR 'dietary supplement':ti,ab,kw OR 'food supplements':ti,ab,kw OR 'food supplement':ti,ab,kw OR 'nutritional supplements':ti,ab,kw OR 'supplementation':ti,ab,kw OR 'supplementations':ti,ab,kw OR 'beta-alanine':ti,ab,kw OR 'beta alanine':ti,ab,kw OR 'β alanine':ti,ab,kw OR 'β-alanine':ti,ab,kw OR 'beta2-alanine':ti,ab,kw OR 'beta2 alanine':ti,ab,kw OR 'B2-alanine':ti,ab,kw OR 'B2 alanine':ti,ab,kw OR 'caffeine':ti,ab,kw OR 'creatine':ti,ab,kw OR 'beetroot':ti,ab,kw OR 'beta vulgaris':ti,ab,kw OR 'sodium bicarbonate':ti,ab,kw OR '$NaHCO_3$':ti,ab,kw OR ' CoQ10':ti,ab,kw OR 'Coenzyme Q10':ti,ab,kw OR 'nitrates':ti,ab,kw OR 'vitamin':ti,ab,kw OR 'beet':ti,ab,kw OR 'beets':ti,ab,kw OR 'protein':ti,ab,kw	4,210,730
	#2	'dietary supplements'/exp	25,215
	#3	#1 OR #2	4,217,133
	#4	'swimming':ti,ab,kw OR 'swimmers':ti,ab,kw OR 'swimmer':ti,ab,kw OR 'swim':ti,ab,kw OR 'front crawl':ti,ab,kw OR 'back crawl':ti,ab,kw OR backstroke:ti,ab,kw OR breaststroke:ti,ab,kw OR 'butterfly stroke':ti,ab,kw OR 'butterfly swimming':ti,ab,kw	59,353
	#5	'swimming'/exp	29,025
	#6	#4 OR #5	65,546
	#7	#3 AND #6	**8117**
PubMed	#1	(((((((((swimming[MeSH Terms]) OR (swimming[Title/Abstract])) OR (swim[Title/Abstract])) OR (swimmer[Title/Abstract])) OR (swimmers[Title/Abstract])) OR (front crawl[Title/Abstract])) OR (back craw[Title/Abstract])) OR (backstroke[Title/Abstract])) OR (breaststroke[Title/Abstract])) OR (butterfly stroke[Title/Abstract])) OR (butterfly swimming[Title/Abstract])	60,431
	#2	((((((((((((((((((((((((((((Dietary supplements[MeSH Terms]) OR (Dietary supplements[Title/Abstract])) OR (Dietary Supplement[Title/Abstract])) OR (Food Supplements[Title/Abstract])) OR (Food Supplement[Title/Abstract])) OR (nutritional supplements[Title/Abstract])) OR (Supplementation[Title/Abstract])) OR (Supplementations[Title/Abstract])) OR (Beta-alanine[Title/Abstract])) OR (Beta alanine[Title/Abstract])) OR (β-alanine[Title/Abstract])) OR (β alanine[Title/Abstract])) OR (Beta2-alanine[Title/Abstract])) OR (Beta2 alanine[Title/Abstract])) OR (B2-alanine[Title/Abstract])) OR (B2 alanine[Title/Abstract])) OR (Caffeine[Title/Abstract])) OR (Creatine[Title/Abstract])) OR (beetroot[Title/Abstract])) OR (beta vulgaris[Title/Abstract])) OR (Sodium Bicarbonate[Title/Abstract])) OR ($NaHCO_3$[Title/Abstract])) OR (CoQ10[Title/Abstract])) OR (Coenzyme Q10[Title/Abstract])) OR (Nitrates[Title/Abstract])) OR	3,562,539

		(Vitamin[Title/Abstract])) OR (Beet[Title/Abstract])) OR (beets[Title/Abstract])) OR (protein[Title/Abstract])) OR (sodium citrate[Title/Abstract])) OR (Carbohydrate[Title/Abstract])	
	#3	#1 AND #2	**6,781**
Web of science	#1	TS = (swimming OR swimmers OR swimmer OR swim OR “front crawl” OR “back crawl” OR backstroke OR breaststroke OR “butterfly stroke” OR “butterfly swimming”) and Preprint Citation Index (Exclude – Database)	81,304
	#2	TS = (“Dietary supplements” OR “Dietary Supplement” OR “Food Supplements” OR “Food Supplement” OR “nutritional supplements” OR Supplementation OR Supplementations OR Beta-alanine OR “Beta alanine” OR β-alanine OR β alanine OR Beta2-alanine OR “Beta2 alanine” OR B2-alanine OR “B2 alanine” OR Caffeine OR Creatine OR beetroot OR “beta vulgaris” OR “Sodium Bicarbonate” OR $NaHCO_3$ OR CoQ10 OR Coenzyme Q10 OR Nitrates OR Vitamin OR Beet OR beets OR protein OR “sodium citrate” OR Carbohydrate) and Preprint Citation Index (Exclude – Database)	5,579,669
	#3	#1 AND #2 and Preprint Citation Index (Exclude – Database)	**10,372**
Cochran Library	#1	MeSH descriptor: [Swimming] explode all trees	651
	#2	(‘Swimming’ OR ‘swimmers’ OR ‘Swimmer’ OR ‘Swim’ OR ‘Front Crawl’ OR ‘Back Crawl’ OR ‘Backstroke’ OR ‘Breaststroke’ OR ‘Butterfly Stroke’ OR ‘Butterfly Swimming’):ti,ab,kw'	1770
	#3	MeSH descriptor: [Dietary Supplements] explode all trees	19,937
	#4	(‘Dietary supplements' OR 'Dietary Supplement' OR 'Food Supplements' OR 'Food Supplement' OR 'nutritional supplements' OR 'Supplementation' OR 'Supplementations' OR 'Beta alanine' OR 'β alanine' OR 'Beta2 alanine' OR 'B2 alanine' OR 'Caffeine' OR 'Creatine' OR 'beetroot ' OR 'beta vulgaris' OR 'Sodium Bicarbonate' OR '$NaHCO_3$' OR 'CoQ10' OR 'Coenzyme Q10' OR 'Nitrates' OR 'Vitamin' OR 'Beet' OR 'beets' OR 'protein'):ti,ab,kw	182,107
	#5	(#1 OR #2) AND (#3 OR #4)	**289**
SPORTDiscus	S1	SU (“Dietary supplements” OR “Dietary Supplement” OR “Food Supplements” OR “Food Supplement” OR “nutritional supplements” OR Supplementation OR Supplementations OR Beta-alanine OR “Beta alanine” OR β-alanine OR β alanine OR Beta2-alanine OR “Beta2 alanine” OR B2-alanine OR “B2 alanine” OR Caffeine OR Creatine OR beetroot OR “beta vulgaris” OR “Sodium Bicarbonate” OR $NaHCO_3$ OR CoQ10 OR Coenzyme Q10 OR Nitrates OR Vitamin OR Beet OR beets OR protein OR “sodium citrate” OR Carbohydrate)	11,359
	S2	SU (swimming OR swimmers OR swimmer OR swim OR "Front Crawl" OR "Back Crawl" OR Backstroke OR Breaststroke OR "Butterfly Stroke" OR "Butterfly Swimming")	13,263
	S3	S1 AND S2	**84**

Table S2. Characteristics of studies included in the review

Study characteristics			Study design	Population characteristics				Intervention characteristics			Outcomes
First author	Year	Countries		Status	Sample size	Gender	Age (year)	Type	Doses	Duration (days)	
Arjmandpanah Eilaki	2018	Iran	RCTs	Trained swimmers	14	M: 14	I: 30.67 ± 6.34<br>C: 32.63 ± 10.26	I: BA<br>C: Placebo	2-3 g/day for 7 days<br>4-6 g/day for next 7 days	14	⑥
Azizi	2011	Iran	RCTs	Trained swimmers	20	F: 20	20.9 ± 1.6	I: Creatine<br>C: Placebo	20 g/day	6	①
Baldassarre	2022	Italy	RCTs	Trained swimmers	8	M:5<br>F:3	23 ± 4	I: CHO<br>C: Placebo	10–12 g/kg of body mass	2	⑥
Burke	1996	Australia	RCTs	Trained swimmers	32	M: 18<br>F: 14	17-25	I: Creatine<br>C: Placebo	20 g/day	5	①②
Campos	2012	Brazil	RCTs	Trained swimmers	10	M: 7<br>F: 3	18.33 ± 3.33	I: $NaHCO_3$<br>C: Placebo	0.3 g/kg of body mass	NA	④
Carbuhn	2018	USA	RCTs	Trained swimmers	17	F: 17	NA	I: Probiotic<br>C: Placebo	4 mg/day	42	②
Chung	2012	Australia	RCTs	Trained swimmers	46	NA	I:21.8 ± 3.3<br>C:21.3 ± 2.4	I: BA<br>C: Placebo	4.8 g/day for 4 weeks<br>3.2 g/day for 6 weeks	70	④⑤
Collomp	1992	France	RCTs	Trained swimmers	14	M: 6<br>F: 8	17±2.1	I: Caffeine<br>C: Placebo	250 mg/kg of body mass	NA	④
Darvishi	2013	Iran	RCTs	Trained swimmers	26	F: 26	I: 16.1 ± 2.5<br>C: 15.7 ± 1.5	I: Quercetin<br>C: Placebo	1000 mg/day	56	④
Dawson	2002	Australia	RCTs	Trained swimmers	20	M: 10<br>F: 10	16.4 ± 1.8	I: Creatine<br>C: Placebo	20 g/day for 5 days<br>5 g/day for 22 days	27	①②④⑦

Table S2. (continued)

Study characteristics			Study design	Population characteristics				Intervention characteristics			
First author	Year	Countries		Status	Sample size	Gender	Age (year)	Type	Dose	Duration (days)	Outcomes
DeSalles Painelli	2013	Brazil.	RCTs	Trained swimmers	14	M: 7<br>F: 7	I: 18.62 ± 1.99<br>C: 20.16 ± 5.91	I1: BA<br>I2: BA_$NaHCO_3$<br>C: Placebo	I1: 3.2 g/day for 1 weeks; 6.4g/day for 4 weeks<br>I2: 3.2 g/day for 7 days; 6.4 g/day for the next 25 days	I1: 35<br>I2: 32	②③
Dubnov-Raz	2015	Israel	RCTs	Trained swimmers	47	NA	NA	I: VD<br>C: Placebo	2000 IU/d	84	①⑥
Emami	2018	Iran	RCTs	Trained swimmers	36	M: 36	17 ± 1	I: CoQ10<br>C: Placebo	300 mg/day	14	④
Esen	2022	UK	RCTs	Trained swimmers	15	M: 10<br>F: 5	25 ± 7	I1: Arg<br>12: Cit<br>C: Placebo	8 g/day	8	②③④
Esen	2023	Turkey	RCDs	Trained swimmers	8	M: 8	25 ± 5	I: Arg<br>C: Placebo	8 g/day	14	②③④
Esen	2019	UK	RCDs	Trained swimmers	10	M: 5<br>F: 5	22 ± 6	I: Beetroot<br>C: Placebo	140 mL/day	6	②③
Gao	1988	USA	RCTs	Trained swimmers	10	M: 10	20.3 ± 0.5	I: $NaHCO_3$<br>C: Placebo	2.9 mmol/kg of body mass	NA	④
Goods	2017	Australia	RCDs	Trained swimmers	9	M: 9	20.8 ± 2.8	I: Caffeine<br>C: Placebo	3 mg/kg of body mass	21	④⑤
Gough	2023	UK	RCDs	Trained swimmers	14	M:14	19 ± 2	I: $NaHCO_3$<br>C: Placebo	0.3 g/kg of body mass	NA	①⑤

Table S2. (continued)

Study characteristics			Study design	Population characteristics				Intervention characteristics			
First author	Year	Countries		Status	Sample size	Gender	Age (year)	Type	Dose	Duration (days)	Outcomes
Grindstaff	1997	USA	RCTs	Trained swimmers	18	M: 7<br>F: 11	15.3 ± 0.6	I: Creatine<br>C: Placebo	21 g/day	9	①②⑦
Hsueh	2018	Taiwan	RCDs	Trained swimmers	16	M: 8<br>F: 8	M: 15.6 ± 1.3<br>F: 15.6 ± 0.9	I: BCAAs_ Cit_Arg<br>C: Placebo	BCAAs: 0.085 g/kg of body mass<br>Cit & Arg: 0.05 g/kg of body mass	NA	①
Joyce	2012	Australia	RCTs	Trained swimmers	8	M: 8	19 ± 3	I: $NaHCO_3$<br>C: Placebo	0.31 g/kg of body mass for 3 days<br>0.1 g/kg of body mass for 1 day	32	③
Juhász	2009	Hungary	RCTs	Trained swimmers	16	M: 16	15.9 ± 1.6	I: Creatine<br>C: Placebo	20 g/day	5	②④⑥
Leenders	1999	USA	RCTs	Trained swimmers	32	M: 18<br>F: 14	F (Creatine): 19.1 ± 0.5;<br>F (Placebo): 19.4 ± 0.8<br>M (Creatine):19.8 ± 0.1<br>M (Placebo): 19.4 ± 0.4	I: Creatine<br>C: Placebo	20 g/day for 6 days<br>10 g/day for 8 days	14	⑦
Lindh	2008	UK	RCTs	Trained swimmers	9	M: 9	20.4 ± 1.7	I: $NaHCO_3$<br>C: Placebo	300 mg/kg of body mass	NA	③④⑤
MacIntosh	1995	Canada	RCDs	Trained swimmers	11	M: 7<br>F: 4	21.8 ± 0.5	I: Caffieine<br>C: Placebo	6 mg/kg of body mass	NA	④
Mendes	2004	Brazil	RCTs	Trained swimmers	18	M: 6<br>F: 12	I: 19.44 ± 2.6<br>C: 19.78 ± 2.33	I: Creatine<br>C: Placebo	20 g/day	8	①②④
Mero	2004	Finland	RCDs	Trained swimmers	16	M: 8<br>F: 8	M: 18.3 ± 0.7<br>F: 17.3 ± 0.8	I: Creatine_$NaHCO_3$<br>C: Placebo	Creatine: 20 g/day for 6 days<br>$NaHCO_3$: 0.3 g/kg of body mass for 1 day	7	④⑤

Table S2. (continued)

Study characteristics			Study design	Population characteristics				Intervention characteristics			
First author	Year	Countries		Status	Sample size	Gender	Age (year)	Type	Dose	Duration (days)	Outcomes
Mero	2013	Finland	RCDs	Trained swimmers	13	M: 13	20.5 ± 1.4	I1: $NaHCO_3$_BA<br>I2: $NaHCO_3$<br>I3: BA<br>C: Placebo	$NaHCO_3$: 0.3 g/kg of body mass for 4 days<br>BA: 4.8 g/day for 28 days	32	②④⑤
Moosakhani	2018	Iran	RCTs	Trained swimmers	24	M: 24	30 ± 5	I: Arg<br>C: Placebo	7 g/day	7	④
Moreno	2023	Spain	RCDs	trained swimmers	13	M: 7<br>F: 6	M: 16.4 ± 1.4<br>F: 15.3 ± 1.75	I: Beetroot<br>C: Placebo	70 ml/day	NA	②④
Moreno-Heredero	2024	Spain	RCDs	Trained swimmers	18	M: 9<br>F: 9	M: 17.00 ± 1.32<br>F: 17.11 ± 1.05	I: Beetroot<br>C: Placebo	70 ml/day	NA	②④⑥
Mujika	1996	France	RCTs	Trained swimmers	20	M: 11<br>F: 9	M: 20.5 ± 2.2<br>F: 19.3 ± 2.8	I: Creatine<br>C: Placebo	20 g/day	5	①②④⑦
Newbury	2022	UK	RCDs	Trained swimmers	8	M: 5<br>F: 3	16-19	I: Caffeine<br>C: Placebo	3 mg/kg of body mass	NA	②④
Peyrebrune	1998	UK	RCTs	Trained swimmers	14	M: 14	I: 20 ± 2<br>C: 21 ± 2	I: Creatine<br>C: Placebo	9 g/day	5	①④⑤⑥
Peyrebrune	2005	ENGLAND	RCTs	Trained swimmers	20	M: 12<br>F: 8	I: 20 ± 1<br>C: 20 ± 2	I: Creatine<br>C: Placebo	20 g/day	10	①④⑤⑥⑦
Pinna	2014	Italy	RCTs	Trained swimmers	14	M: 14	34.7 ± 7.5	I: Beetroot<br>C: Placebo	0.5 L/day	6	⑥
Pospieszna	2016	Poland	RCDs	Trained swimmers	11	F: 11	20.9 ± 1.3	I: Beetroot<br>C: Placebo	5.1 mmol/L/day	8	⑥

Table S2. (continued)

Study characteristics			Study design	Population characteristics				Intervention characteristics			
First author	Year	Countries		Status	Sample size	Gender	Age (year)	Type	Dose	Duration (days)	Outcomes
Pruscino	2008	Australia	RCTs	Trained swimmers	6	M: 6	NA	I1: Caffeine_$NaHCO_3$<br>I2: Caffeine<br>I3: $NaHCO_3$<br>C: Placebo	Caffeine: 6.2±0.3 mg/kg of body mass<br>$NaHCO_3$: 0.3 g/kg of body mass	4	③④⑤
Reilly	1999	UK	RCTs	Trained swimmers	7	M: 4<br>F: 3	14.2	I: CHO<br>C: Placebo	NA	3	②③④
Rockwell	2020	USA	RCTs	Trained swimmers	19	NA	I: 20.1 ± 1.6<br>C: 19.7 ± 0.8	I: VD<br>C: Placebo	5000 IU/day	84	⑦
Rondanelli	2020	Italy	RCTs	Trained swimmers	27	M: 20<br>F: 7	I: 31.86 ± 8.06<br>C: 30.23 ± 5.10	I: RG<br>C: Placebo	25 g/day	35	①③④⑦
Roshan	2013	Iran	RCTs	Trained swimmers	16	M: 16	19 ± 4	I: Creatine<br>C: Placebo	20 g/day	6	④
Russell	2014	Canada	RCDs	Trained swimmers	10	M: 10	14.9 ± 0.4	I: CSC<br>C: Placebo	0.5 g/kg of body mass	5	④
Salgueiro	2022	Brazil.	RCTs	Trained swimmers	10	M: 10	18.2 ± 1.7	I: Caffeine<br>C: Placebo	6 mg/kg of body mass	8	④
Saritas	2011	Turkey	RCTs	Trained swimmers	40	M: 40	13-25	I: Royal jelly<br>C: Placebo	2 g/day	28	⑦
Schulz	1989	USA	RCDs	Trained swimmers	20	M: 13<br>F: 7	18.9	I: CHO<br>C: Placebo	12 g/day	12	③
Scorcine	2013	Brazil,	RCTs	Trained swimmers	30	M: 30	29.20 ± 7.76	I: Creatine<br>C: Placebo	5 g/day	63	①

Table S2. (continued)

Study characteristics			Study design	Population characteristics				Intervention characteristics			
First author	Year	Countries		Status	Sample size	Gender	Age (year)	Type	Dose	Duration (days)	Outcomes
Selsby	2003	USA	RCTs	Trained swimmers	15	M: 8<br>F: 7	I: 18.8 ± 0.25<br>C: 19.75 ± 0.37	I: Creatine<br>C: Placebo	0.3 g/kg of body mass for 5 days<br>2.25 g/day for 9 days	14	①②⑦
Sharman	1971	UK	RCTs	Trained swimmers	15	M: 15	I: 16.03 ± 1.28<br>C: 15.57 ± 1.17	I: VE<br>C: Placebo	400 mg/day	42	⑥⑦
Siegler	2010	UK	RCTs	Trained swimmers	14	M: 6<br>F: 8	NA	I: $NaHCO_3$<br>C: Placebo	0.3 g/kg of body mass	NA	④⑤
Silva	2007	Portugal	RCTs	Trained swimmers	16	F: 16	I: 16.3 ± 1.8<br>C: 15.7 ± 1.2	I: Creatine<br>C: Placebo	20 g/day	21	⑦
Theodorou	1999	UK	RCTs	Trained swimmers	22	M: 12<br>F: 10	M: 19.7 ± 2.3<br>F: 17.7 ± 2	I: Creatine<br>C: Placebo	25 g/day for 4 days	4	①②⑦
Thompson	1996	UK	RCTs	Trained swimmers	10	F: 10	NA	I: Creatine<br>C: Placebo	2 g/day	42	②
Trappe	1994	USA	RCTs	Trained swimmers	20	M: 20	20.1 ± 0.6	I: Carn<br>C: Placebo	4 g/day	7	④⑤
Vatani	2011	Iran	RCTs	Trained swimmers	20	M: 20	I: 21.32 ± 2.74<br>C: 21.32 ± 2.74	I: Creatine<br>C: Placebo	20 g/day	6	①②
Wu	2023	Philippines	RCTs	Trained swimmers	20	M: 20	23.1 ± 2.7	I: Protein<br>C: CHO	Protein: 0.5 g/kg of body mass<br>CHO: 0.5 g/kg of body mass	14	②⑥
Zajac	2009	Poland	RCTs	Trained swimmers	8	M: 8	15.1 ± 0.4	I: $NaHCO_3$<br>C: Placebo	300 mg/kg of body mass	NA	⑤

Note: RCTs: randomized controlled trials, RCDs: randomized crossover designs, M:male, F: female, NA: not available, I: intervention, C: control, BA: Beta-alanine, CHO: Carbohydrate, $NaHCO_3$: Sodium bicarbonate, Carn: L-Carnitine, VE: Vitamin E, RG: Rice germ, CSC: chronic sodium citrate, VD: Vitamin D_3, Arg: L-arginine, Cit: L-Citrulline, BCAAs: Branched-Chain Amino Acids, CoQ10: Coenzyme Q10 , _: combined with, ①50 m time, ②100 m time, ③200 m time, ④blood lactate, ⑤blood pH, ⑥heart rate, ⑦body mass

Figure S1. Network of intervention comparisons. a: blood lactate, b: blood pH, c: heart rate, d: body mass

a

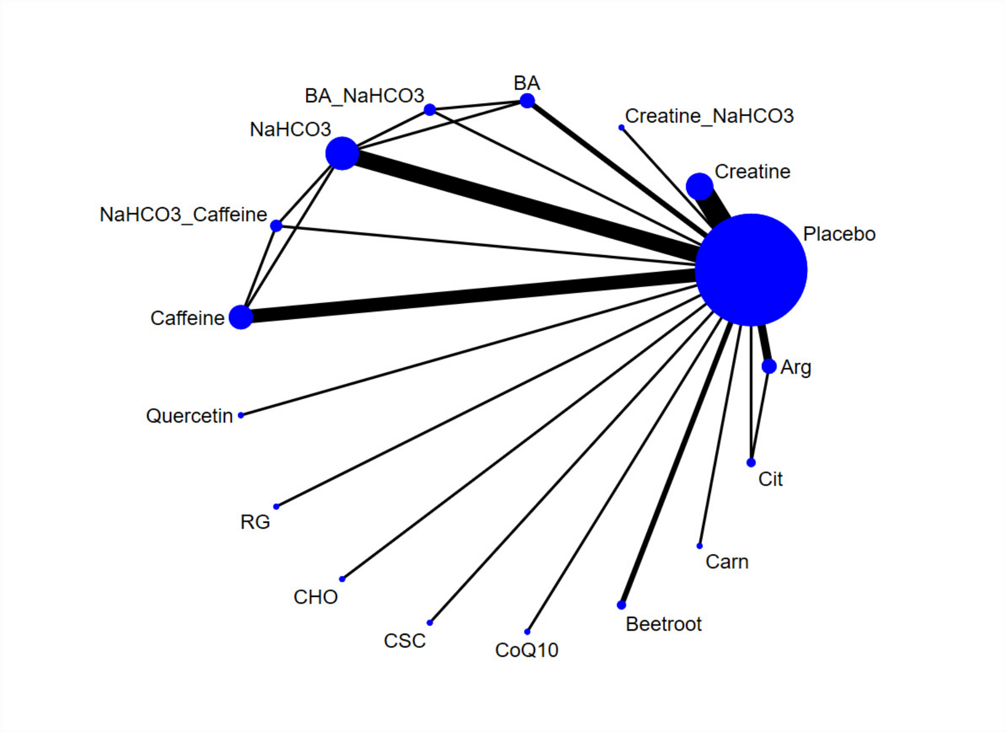

b

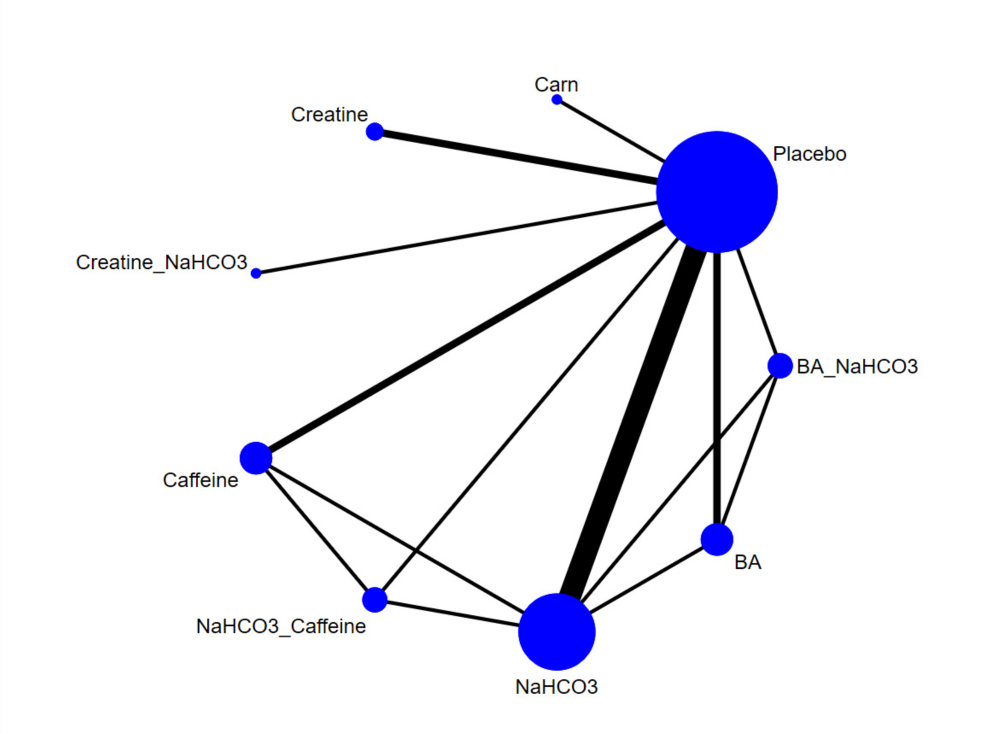

Figure S1. (continued)

c

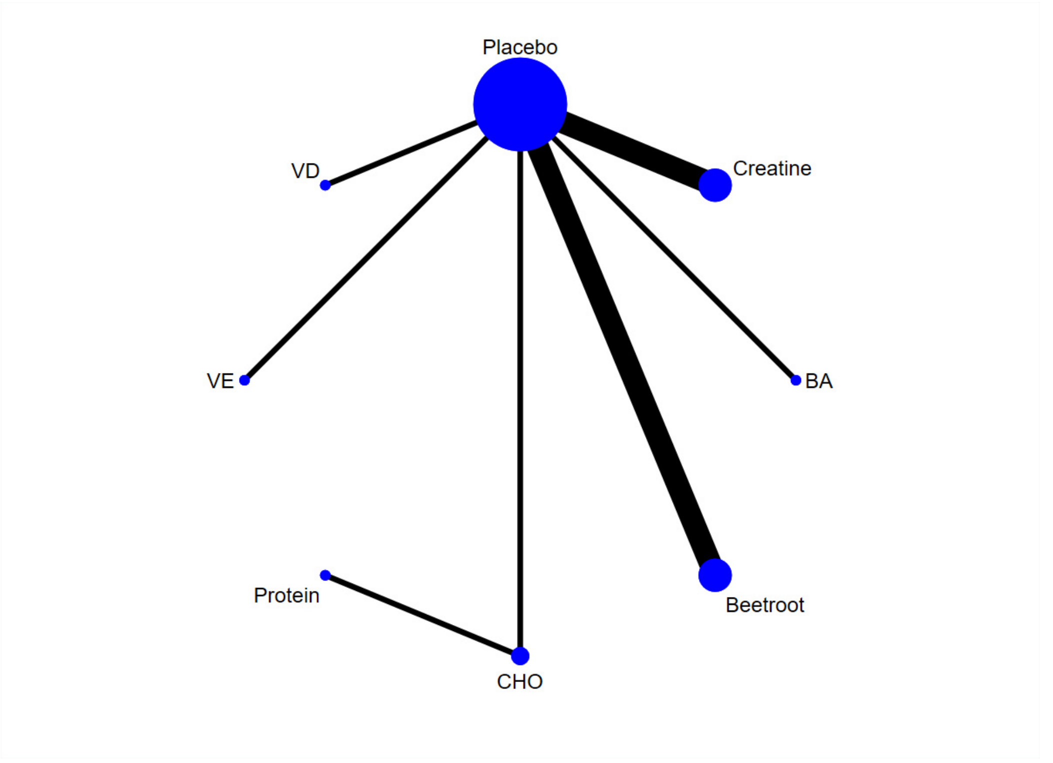

d

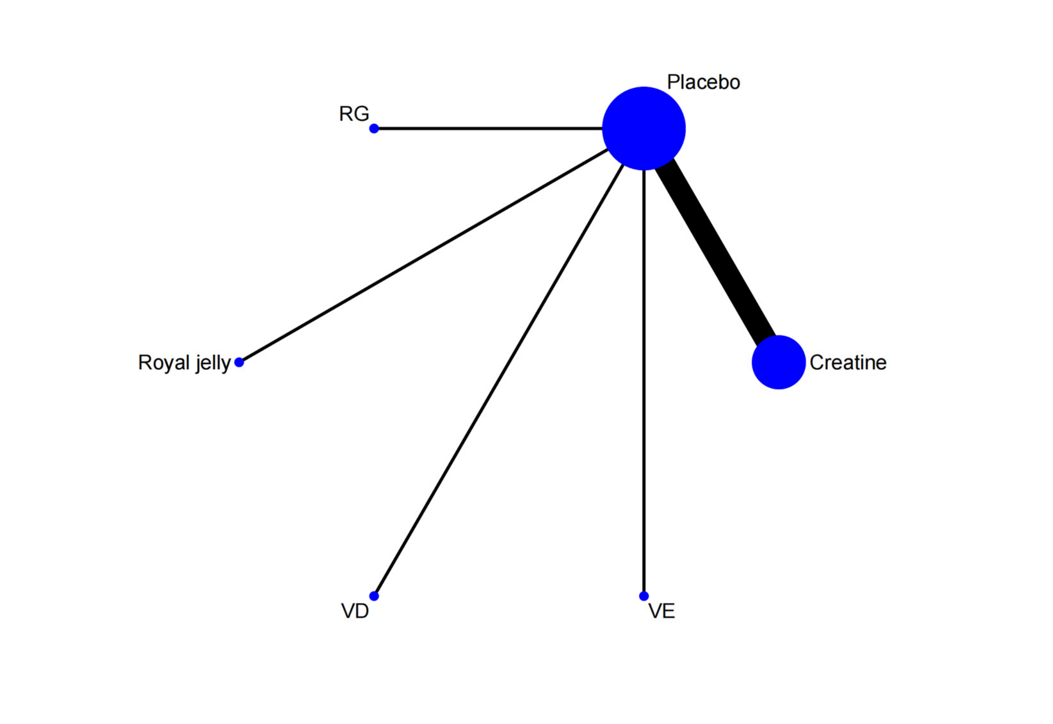

Note: BA: Beta-alanine, CHO: Carbohydrate, $NaHCO_3$: Sodium bicarbonate, Carn: L-Carnitine, VE: Vitamin E, RG: Rice germ, CSC: Chronic sodium citrate, VD: Vitamin D_3, Arg: L-arginine, Cit: L-Citrulline, BCAAs: Branched-Chain Amino Acids, CoQ10: Coenzyme Q10

Table S3. League table of swimming performance

Table S3.1. League table of swimming performance for blood lactate

Arg	-	-	-	-	-	-	-	-	-	-	-	-	-	-	-	-
0.84 (-0.09, 1.77)	BA	-	-	-	-	-	-	-	-	-	-	-	-	-	-	-
0.23 (-0.81, 1.26)	-0.62 (-1.44, 0.22)	BA_NaHCO$_3$	-	-	-	-	-	-	-	-	-	-	-	-	-	-
0.91 (-0.17, 2)	0.06 (-0.95, 1.13)	0.67 (-0.43, 1.85)	Beetroot	-	-	-	-	-	-	-	-	-	-	-	-	-
-0.14 (-1.02, 0.77)	-0.97 (-1.81, -0.15)	-0.36 (-1.3, 0.58)	-1.04 (-2.08, -0.05)	Caffeine	-	-	-	-	-	-	-	-	-	-	-	-
-0.84 (-2.22, 0.55)	-1.68 (-3.02, -0.33)	-1.07 (-2.49, 0.36)	-1.76 (-3.22, -0.29)	-0.71 (-2.03, 0.64)	Carn	-	-	-	-	-	-	-	-	-	-	-
0.62 (-1.27, 2.41)	-0.23 (-2.05, 1.55)	0.39 (-1.49, 2.25)	-0.3 (-2.19, 1.56)	0.74 (-1.07, 2.52)	1.45 (-0.63, 3.5)	CHO	-	-	-	-	-	-	-	-	-	-
0.88 (-0.18, 1.96)	0.04 (-1.17, 1.28)	0.66 (-0.66, 1.98)	-0.03 (-1.37, 1.3)	1.01 (-0.18, 2.22)	1.72 (0.07, 3.36)	0.27 (-1.7, 2.3)	Cit	-	-	-	-	-	-	-	-	-
0.9 (-0.4, 2.22)	0.06 (-1.21, 1.36)	0.67 (-0.67, 2.04)	-0.01 (-1.42, 1.37)	1.03 (-0.22, 2.31)	1.75 (0.09, 3.37)	0.28 (-1.73, 2.36)	0.02 (-1.51, 1.54)	CoQ10	-	-	-	-	-	-	-	-
0.67 (-0.12, 1.47)	-0.17 (-0.9, 0.56)	0.44 (-0.41, 1.32)	-0.24 (-1.18, 0.67)	0.8 (0.11, 1.5)	1.51 (0.25, 2.77)	0.05 (-1.65, 1.82)	-0.21 (-1.37, 0.92)	-0.23 (-1.42, 0.96)	Creatine	-	-	-	-	-	-	-
-1.81 (-3.47, -0.13)	-2.66 (-4.29, -0.98)	-2.04 (-3.76, - 0.32)	-2.72 (-4.46, -0.98)	-1.68 (-3.28, -0.03)	-0.95 (-2.96, 0.96)	-2.42 (-4.69, -0.09)	-2.69 (-4.52, -0.85)	-2.72 (-4.57, -0.81)	-2.48 (-4.05, -0.89)	Creatine_NaHCO$_3$	-	-	-	-	-	-

Table S3.1. (continued)

0.81 (-0.74, 2.36)	-0.03 (-1.58, 1.48)	0.57 (-1.01, 2.18)	-0.09 (-1.78, 1.51)	0.94 (-0.58, 2.44)	1.65 (-0.21, 3.45)	0.19 (-2, 2.41)	-0.07 (-1.81, 1.65)	-0.09 (-1.92, 1.69)	0.14 (-1.31, 1.56)	2.6 (0.54, 4.66)	CSC	-	-	-	-	-
0 (-0.82, 0.83)	-0.84 (-1.52, -0.16)	-0.22 (-1.01, 0.57)	-0.89 (-1.88, 0.03)	0.14 (-0.52, 0.79)	0.85 (-0.44, 2.11)	-0.61 (-2.35, 1.17)	-0.88 (-2.04, 0.27)	-0.89 (-2.12, 0.3)	-0.66 (-1.27, -0.07)	1.81 (0.22, 3.39)	-0.8 (-2.27, 0.66)	$NaHCO_3$	-	-	-	-
-1.01 (-2.1, 0.06)	-1.85 (-2.86, -0.84)	-1.24 (-2.32, -0.13)	-1.91 (-3.12, -0.75)	-0.87 (-1.75, 0)	-0.17 (-1.62, 1.28)	-1.61 (-3.5, 0.31)	-1.89 (-3.24, -0.58)	-1.9 (-3.32, -0.53)	-1.67 (-2.6, -0.78)	0.8 (-0.96, 2.56)	-1.81 (-3.43, -0.21)	-1.01 (-1.86, -0.16)	$NaHCO_3$_Caffeine	-	-	-
0.61 (-0.08, 1.31)	-0.23 (-0.85, 0.4)	0.38 (-0.38, 1.17)	-0.3 (-1.15, 0.52)	0.74 (0.17, 1.32)	1.45 (0.26, 2.65)	0 (-1.67, 1.72)	-0.28 (-1.34, 0.79)	-0.29 (-1.43, 0.84)	-0.06 (-0.46, 0.33)	2.42 (0.89, 3.94)	-0.2 (-1.57, 1.22)	0.6 (0.16, 1.07)	1.61 (0.81, 2.46)	Placebo	-	-
0.34 (-0.84, 1.57)	-0.49 (-1.64, 0.68)	0.12 (-1.11, 1.38)	-0.56 (-1.87, 0.73)	0.48 (-0.65, 1.64)	1.18 (-0.35, 2.75)	-0.26 (-2.21, 1.73)	-0.54 (-1.98, 0.92)	-0.55 (-2.04, 0.95)	-0.32 (-1.37, 0.75)	2.16 (0.35, 3.96)	-0.46 (-2.15, 1.25)	0.34 (-0.72, 1.45)	1.35 (0.09, 2.66)	-0.26 (-1.24, 0.73)	Quercetin	-
0.21 (-0.98, 1.41)	-0.64 (-1.77, 0.54)	-0.02 (-1.28, 1.24)	-0.71 (-2, 0.58)	0.34 (-0.79, 1.51)	1.04 (-0.47, 2.62)	-0.41 (-2.35, 1.56)	-0.68 (-2.13, 0.81)	-0.69 (-2.18, 0.81)	-0.46 (-1.52, 0.6)	2.02 (0.16, 3.86)	-0.61 (-2.28, 1.09)	0.2 (-0.86, 1.3)	1.21 (-0.03, 2.53)	-0.4 (-1.37, 0.59)	-0.15 (-1.53, 1.26)	RG

Note: BA: Beta-alanine, CHO: Carbohydrate, $NaHCO_3$: Sodium bicarbonate, Carn: L-Carnitine, RG: Rice germ, CSC: Chronic sodium citrate, Arg: L-arginine, Cit: L-Citrulline, CoQ10: Coenzyme Q10

Table S3.2. League table of swimming performance for blood pH

BA	-	-	-	-	-	-	-	-
-1.13 (-1.98, -0.31)	BA_$NaHCO_3$	-	-	-	-	-	-	-
-0.5 (-1.4, 0.43)	0.64 (-0.36, 1.66)	Caffeine	-	-	-	-	-	-
0.31 (-0.88, 1.51)	1.46 (0.15, 2.73)	0.82 (-0.46, 2.06)	Carn	-	-	-	-	-
-0.12 (-1.08, 0.88)	1.02 (-0.1, 2.14)	0.39 (-0.69, 1.44)	-0.43 (-1.72, 0.87)	Creatine	-	-	-	-
-3.84 (-5.96, -1.83)	-2.69 (-4.86, -0.66)	-3.35 (-5.5, -1.3)	-4.16 (-6.41, -2.01)	-3.74 (-5.89, -1.62)	Creatine_$NaHCO_3$	-	-	-
-0.99 (-1.65, -0.33)	0.15 (-0.62, 0.93)	-0.49 (-1.25, 0.25)	-1.3 (-2.46, -0.18)	-0.87 (-1.78, 0.03)	2.85 (0.88, 4.91)	$NaHCO_3$	-	-
-0.72 (-1.69, 0.23)	0.42 (-0.65, 1.48)	-0.23 (-1.09, 0.63)	-1.04 (-2.33, 0.26)	-0.61 (-1.71, 0.51)	3.11 (1.01, 5.3)	0.26 (-0.53, 1.05)	$NaHCO_3$_Caffeine	-
-0.05 (-0.65, 0.56)	1.09 (0.32, 1.85)	0.45 (-0.29, 1.15)	-0.37 (-1.4, 0.67)	0.06 (-0.71, 0.83)	3.79 (1.85, 5.8)	0.93 (0.49, 1.39)	0.67 (-0.11, 1.46)	Placebo

Note: BA: Beta-alanine, $NaHCO_3$: Sodium bicarbonate, Carn: L-Carnitine

Table S3.3. League table of swimming performance for heart rate

BA	-	-	-	-	-	-	-
0.2 (-1.09, 1.52)	Beetroot	-	-	-	-	-	-
-0.39 (-2.3, 1.56)	-0.59 (-2.23, 1.06)	CHO	-	-	-	-	-
0.11 (-1.18, 1.4)	-0.09 (-0.89, 0.71)	0.51 (-1.12, 2.12)	Creatine	-	-	-	-
0.2 (-0.96, 1.38)	0 (-0.57, 0.58)	0.6 (-0.94, 2.11)	0.09 (-0.45, 0.64)	Placebo	-	-	-
-0.61 (-2.77, 1.53)	-0.81 (-2.71, 1.1)	-0.22 (-1.24, 0.79)	-0.73 (-2.61, 1.16)	-0.81 (-2.62, 1)	Protein	-	-
0.21 (-1.18, 1.61)	0.01 (-0.96, 0.98)	0.6 (-1.12, 2.29)	0.1 (-0.85, 1.04)	0 (-0.77, 0.77)	0.81 (-1.16, 2.78)	VD	-
0.09 (-1.55, 1.72)	-0.11 (-1.4, 1.16)	0.48 (-1.46, 2.37)	-0.02 (-1.29, 1.24)	-0.11 (-1.26, 1.01)	0.7 (-1.46, 2.82)	-0.11 (-1.52, 1.24)	VE

Note: BA: Beta-alanine, CHO: Carbohydrate, VE: Vitamin E, VD: Vitamin D_3

Table S3.4. League table of swimming performance for body mass

Creatine	-	-	-	-	-
0.01 (-0.74, 0.68)	Placebo	-	-	-	-
-0.17 (-2.14, 1.72)	-0.18 (-1.97, 1.62)	RG	-	-	-
-0.26 (-2.27, 1.7)	-0.27 (-2.1, 1.59)	-0.09 (-2.65, 2.49)	Royal jelly	-	-
0.16 (-1.87, 2.1)	0.15 (-1.71, 2.01)	0.33 (-2.26, 2.89)	0.41 (-2.22, 3.03)	VD	-
-0.42 (-2.51, 1.57)	-0.43 (-2.35, 1.48)	-0.25 (-2.89, 2.36)	-0.16 (-2.82, 2.48)	-0.58 (-3.27, 2.1)	VE

Note: BA: VE: Vitamin E, RG: Rice germ, VD: Vitamin D_3

Figure S2. Ranking of nutritional supplement interventions based on the probability of their effects. a: blood lactate, b: blood pH, c: heart rate, d: body mass

a

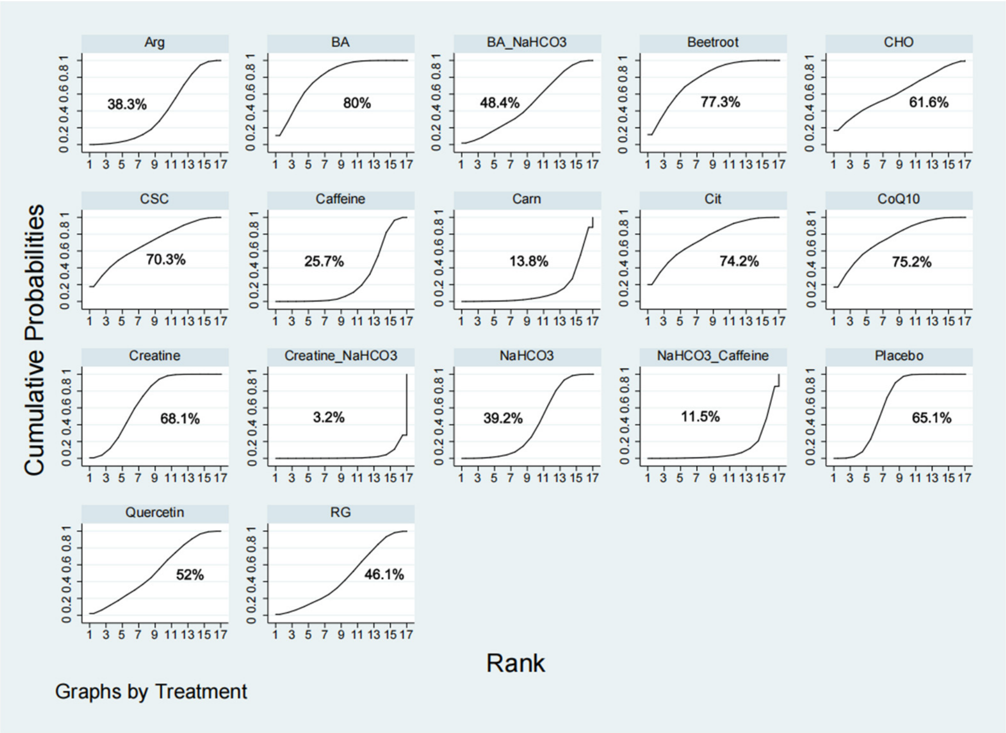

b

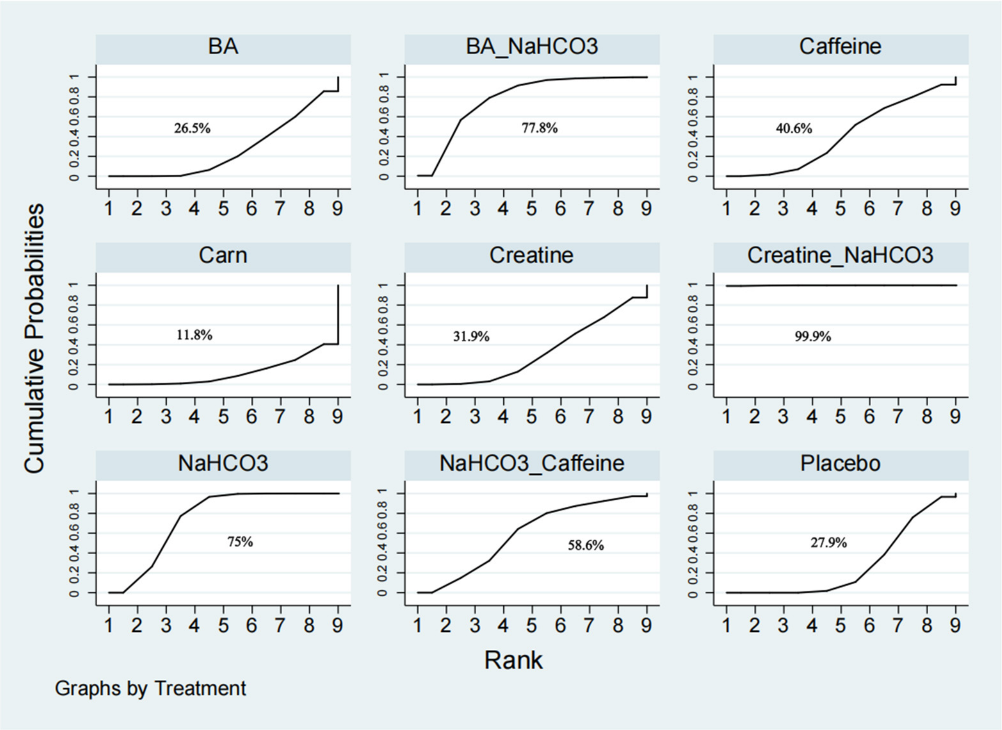

Figure S2. (continued)

c

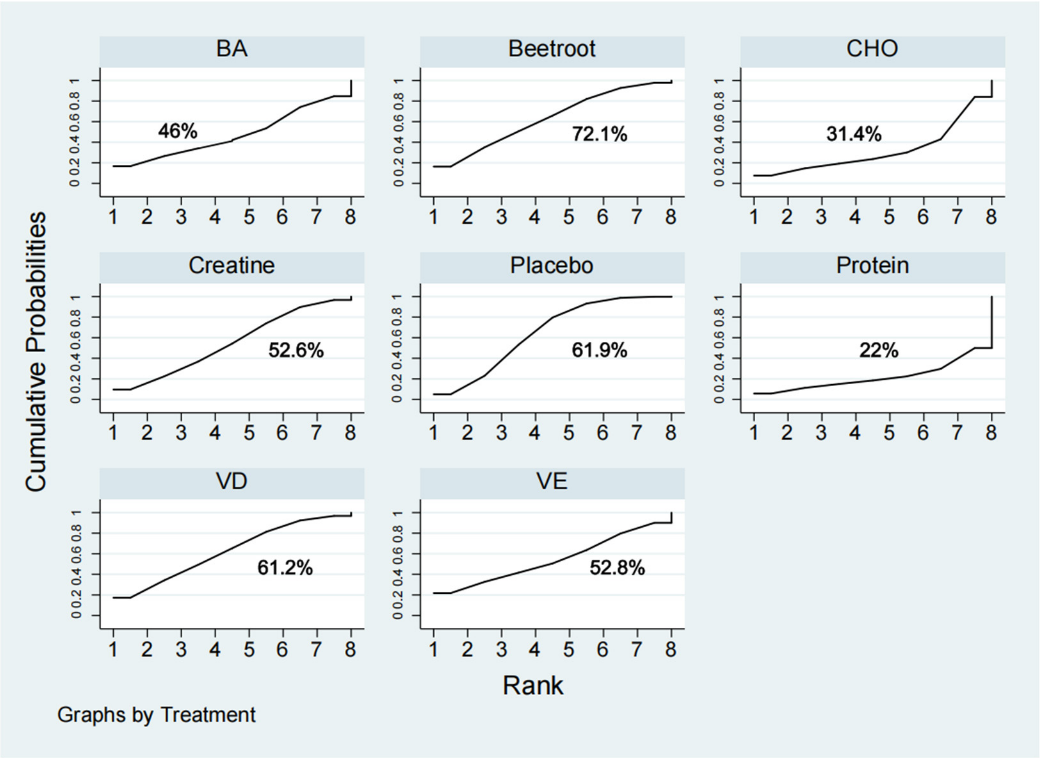

d

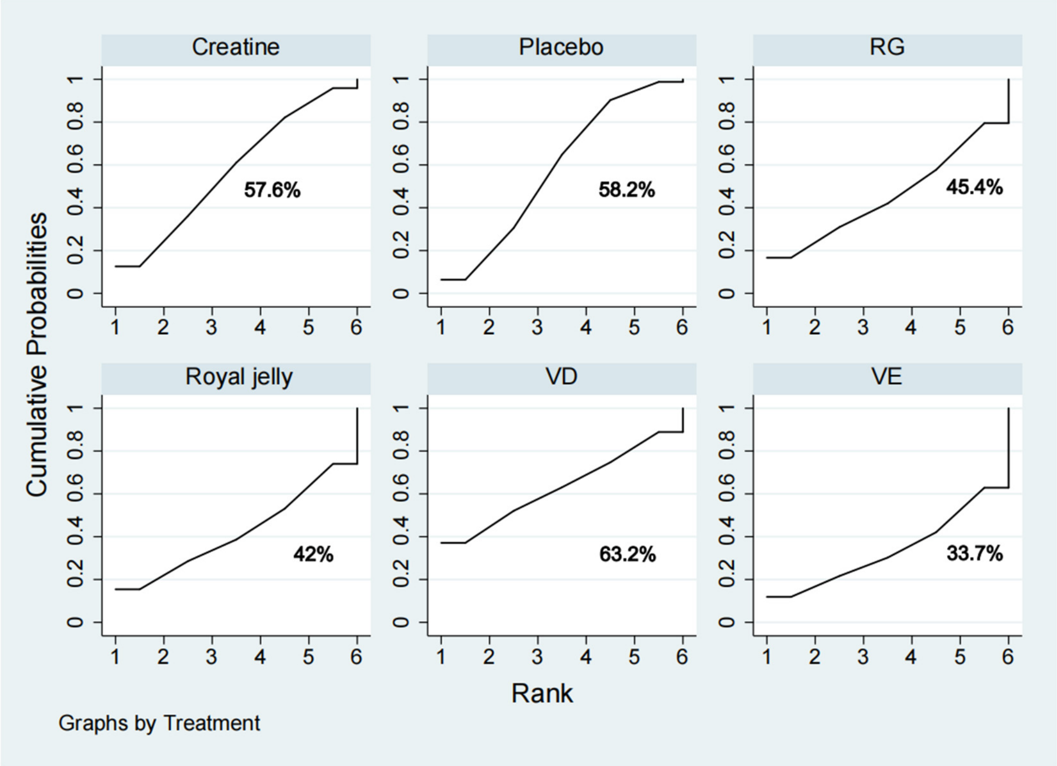

Note: BA: Beta-alanine, CHO: Carbohydrate, $NaHCO_3$: Sodium bicarbonate, Carn: L-Carnitine, VE: Vitamin E, RG: Rice germ, CSC: Chronic sodium citrate, VD: Vitamin D_3, Arg: L-arginine, Cit: L-Citrulline, BCAAs: Branched-Chain Amino Acids, CoQ10: Coenzyme Q10

Figure S3. Risk of bias

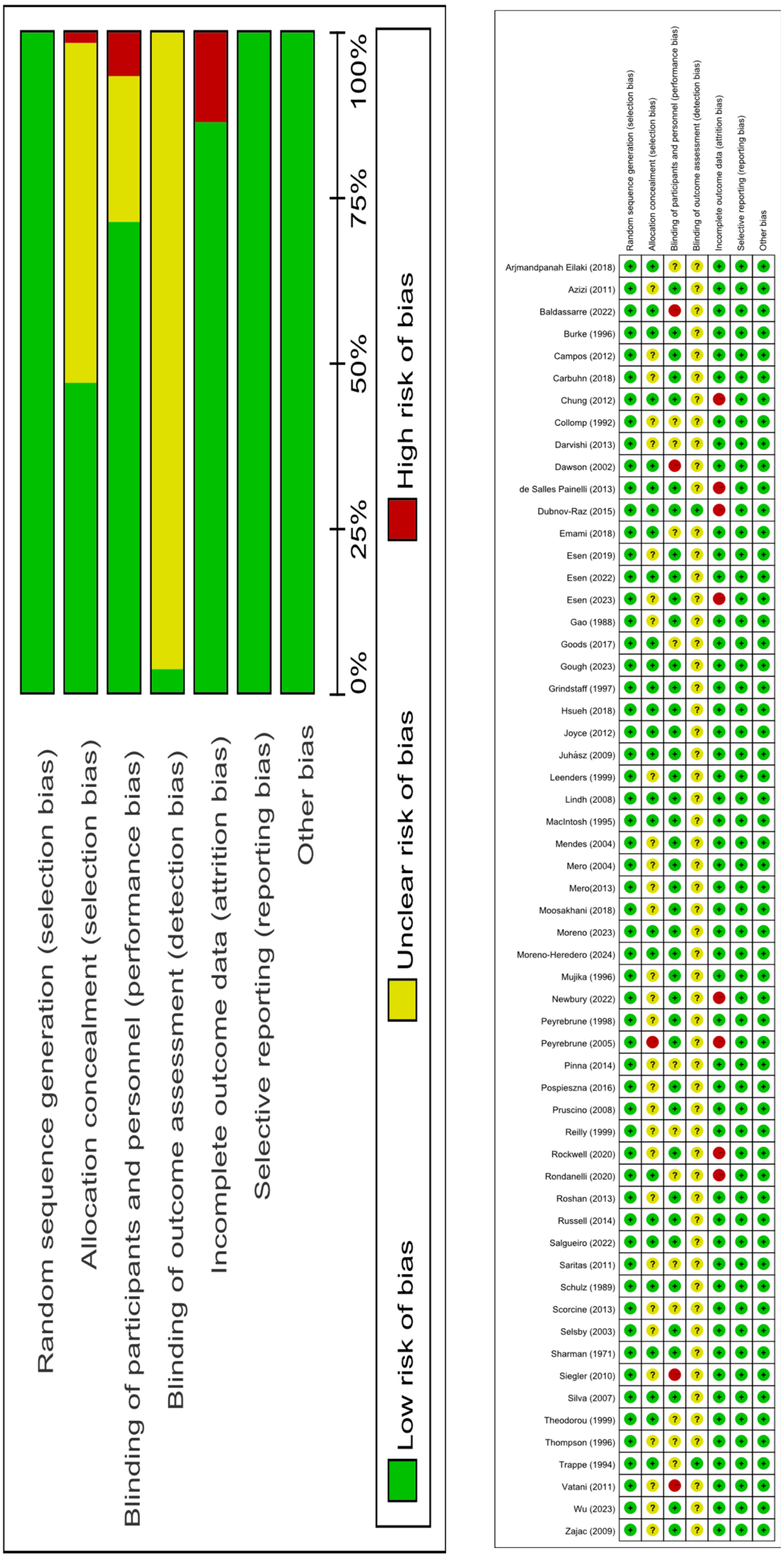

Table S4. Overall risk of bias assessment for each study

Study	Random sequence generation	Allocation concealment	Blinding of participants and personnel	Blinding of outcome assessment	Incomplete outcome data	Selective reporting	Other bias	Overall
Arjmandpanah Eilaki (2018)	Low	Low	Unclear	Unclear	Low	Low	Low	unclear
Azizi (2011)	Low	Unclear	Low	Unclear	Low	Low	Low	unclear
Baldassarre (2022)	Low	Low	High	Unclear	Low	Low	Low	High
Burke (1996)	Low	Low	Low	Unclear	Low	Low	Low	unclear
Campos (2012)	Low	Unclear	Low	Unclear	Low	Low	Low	unclear
Carbuhn (2018)	Low	Unclear	Low	Unclear	Low	Low	Low	unclear
Chung (2012)	Low	Low	Low	Unclear	High	Low	Low	High
Collomp (1992)	Low	Unclear	Unclear	Unclear	Low	Low	Low	unclear
Darvishi (2013)	Low	Unclear	Unclear	Unclear	Low	Low	Low	unclear
Dawson (2002)	Low	Low	High	Unclear	Low	Low	Low	High
de Salles Painelli (2013)	Low	Low	Low	Unclear	High	Low	Low	High
Dubnov-Raz (2015)	Low	Low	Low	Low	High	Low	Low	High
Emami (2018)	Low	Low	Unclear	Unclear	Low	Low	Low	unclear
Essen (2019)	Low	Unclear	Low	Unclear	Low	Low	Low	unclear
Esen (2022)	Low	Low	Low	Unclear	Low	Low	Low	unclear
Esen (2023)	Low	Unclear	Low	Unclear	High	Low	Low	High
Gao (1988)	Low	Unclear	Low	Unclear	Low	Low	Low	unclear
Goods (2017)	Low	Low	Unclear	Unclear	Low	Low	Low	unclear
Gough (2023)	Low	Low	Low	Unclear	Low	Low	Low	unclear
Grindstaff (1997)	Low	Low	Low	Unclear	Low	Low	Low	unclear
Hsueh (2018)	Low	Low	Low	Unclear	Low	Low	Low	unclear
Joyce (2012)	Low	Low	Low	Unclear	Low	Low	Low	unclear

Table S4. (continued)

Study	Random sequence generation	Allocation concealment	Blinding of participants and personnel	Blinding of outcome assessment	Incomplete outcome data	Selective reporting	Other bias	Overall
Juhász (2009)	Low	Low	Low	Unclear	Low	Low	Low	unclear
Leenders (1999)	Low	Unclear	Low	Unclear	Low	Low	Low	unclear
Lindh (2008)	Low	Low	Low	Unclear	Low	Low	Low	unclear
MacIntosh (1995)	Low	Low	Low	Unclear	Low	Low	Low	unclear
Mendes (2004)	Low	Unclear	Low	Unclear	Low	Low	Low	unclear
Mero (2004)	Low	Unclear	Low	Unclear	Low	Low	Low	unclear
Mero (2013)	Low	Unclear	Low	Unclear	Low	Low	Low	unclear
Moosakhani (2018)	Low	Unclear	Low	Unclear	Low	Low	Low	unclear
Moreno (2023)	Low	Low	Low	Unclear	Low	Low	Low	unclear
Moreno-Heredero (2024)	Low	Low	Low	Unclear	Low	Low	Low	unclear
Mujika (1996)	Low	Unclear	Low	Unclear	Low	Low	Low	unclear
Newbury (2022)	Low	Unclear	Low	Unclear	High	Low	Low	High
Peyrebrune (1998)	Low	Unclear	Low	Unclear	Low	Low	Low	unclear
Peyrebrune (2005)	Low	High	Low	Unclear	High	Low	Low	High
Pinna (2014)	Low	Unclear	Unclear	Unclear	Low	Low	Low	unclear
Pospieszna (2016)	Low	Unclear	Low	Unclear	Low	Low	Low	unclear
Pruscino (2008)	Low	Unclear	Low	Unclear	Low	Low	Low	unclear
Reilly (1999)	Low	Unclear	Unclear	Unclear	Low	Low	Low	unclear
Rockwell (2020)	Low	Unclear	Low	Unclear	High	Low	Low	High
Rondanelli (2020)	Low	Low	Unclear	Unclear	High	Low	Low	High
Roshan (2013)	Low	Unclear	Low	Unclear	Low	Low	Low	unclear
Russell (2014)	Low	Low	Low	Unclear	Low	Low	Low	unclear

Table S4. (continued)

Study	Random sequence generation	Allocation concealment	Blinding of participants and personnel	Blinding of outcome assessment	Incomplete outcome data	Selective reporting	Other bias	Overall
Salgueiro (2022)	Low	Low	Low	Unclear	Low	Low	Low	unclear
Saritas (2011)	Low	Unclear	Unclear	Unclear	Low	Low	Low	unclear
Schulz (1989)	Low	Low	Low	Unclear	Low	Low	Low	unclear
Scorcine (2013)	Low	Unclear	Unclear	Unclear	Low	Low	Low	unclear
Selsby (2003)	Low	Unclear	Low	Unclear	Low	Low	Low	unclear
Sharman (1971)	Low	Low	Low	Unclear	Low	Low	Low	unclear
Siegler (2010)	Low	Unclear	High	Unclear	Low	Low	Low	High
Silva (2007)	Low	Low	Low	Unclear	Low	Low	Low	unclear
Theodorou (1999)	Low	Low	Unclear	Unclear	Low	Low	Low	unclear
Thompson (1996)	Low	Unclear	Unclear	Unclear	Low	Low	Low	unclear
Trappe (1994)	Low	Low	Unclear	Low	Low	Low	Low	unclear
Vatani (2011)	Low	Unclear	High	Unclear	Low	Low	Low	High
Wu (2023)	Low	Unclear	Low	Unclear	Low	Low	Low	unclear
Zajac (2009)	Low	Unclear	Low	Unclear	Low	Low	Low	unclear

Note: The overall risk of bias for each study was assessed conservatively. A study was rated as high risk if any domain was judged as "high risk." If at least one domain was deemed "unclear", the study was categorized as having an unclear risk. Only studies with all domains rated as "low risk" were classified as having a low overall risk of bias.

Figure S4. Publication bias for a: 50 m time, b:100m time, c: 200 m time, d: blood lactate, e: blood pH, f: heart rate, g: body mass

a

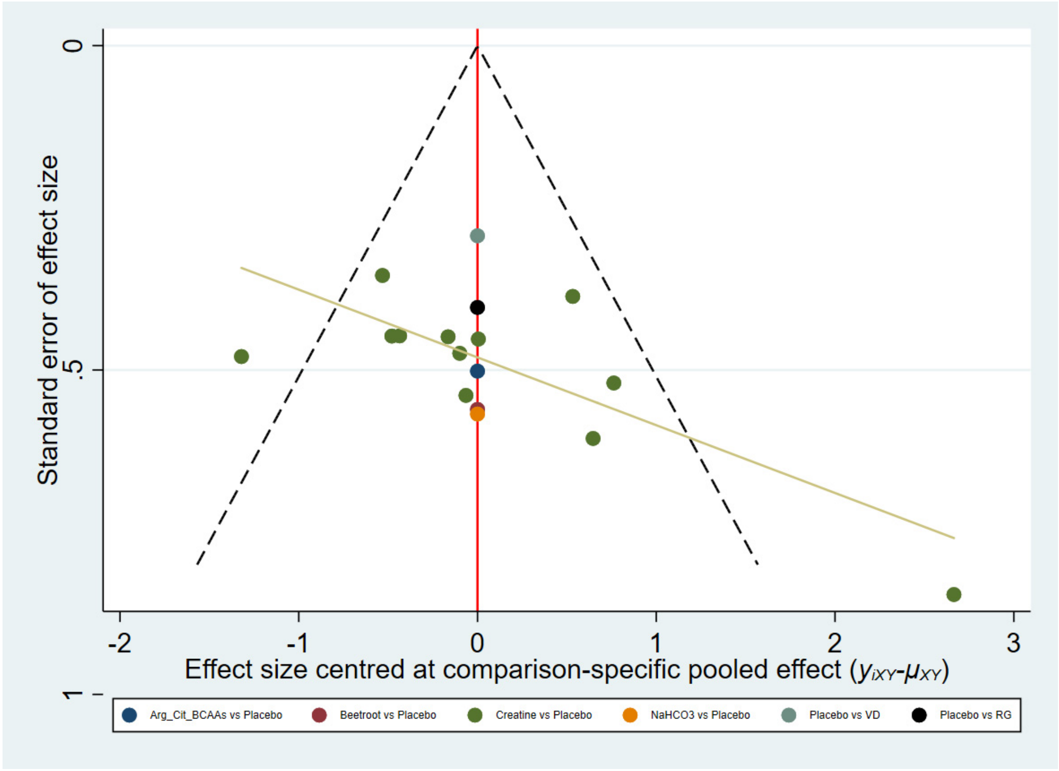

b

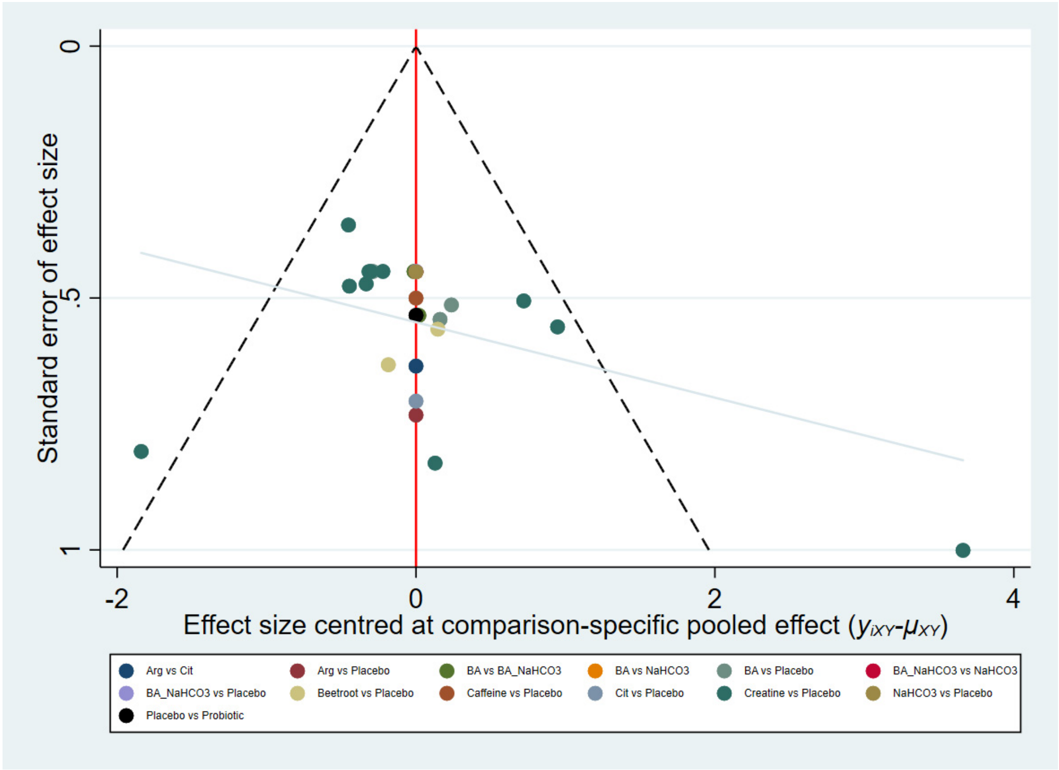

Figure S4. (continued)

c

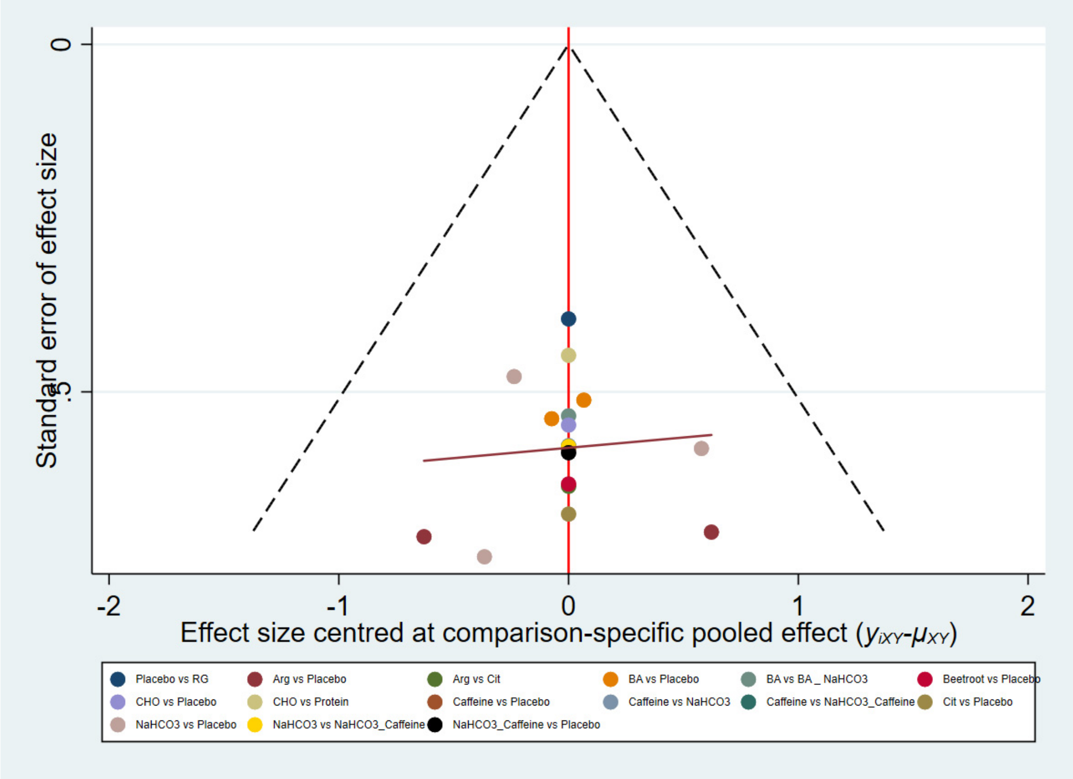

d

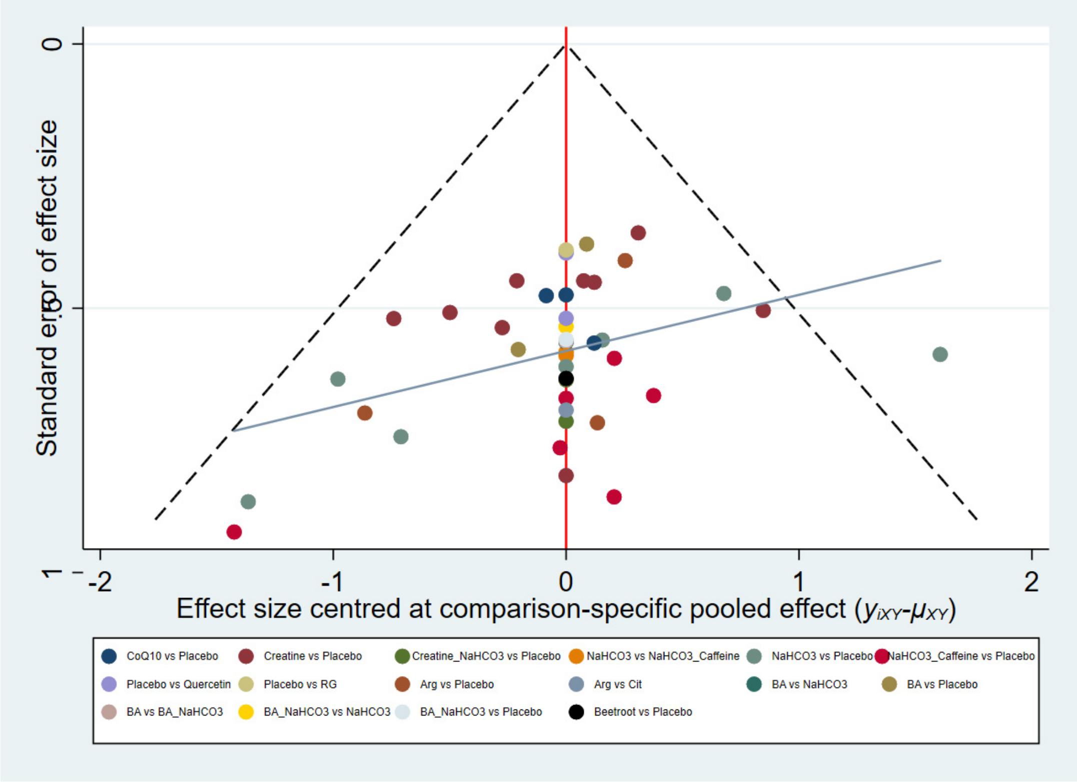

Figure S4. (continued)

e

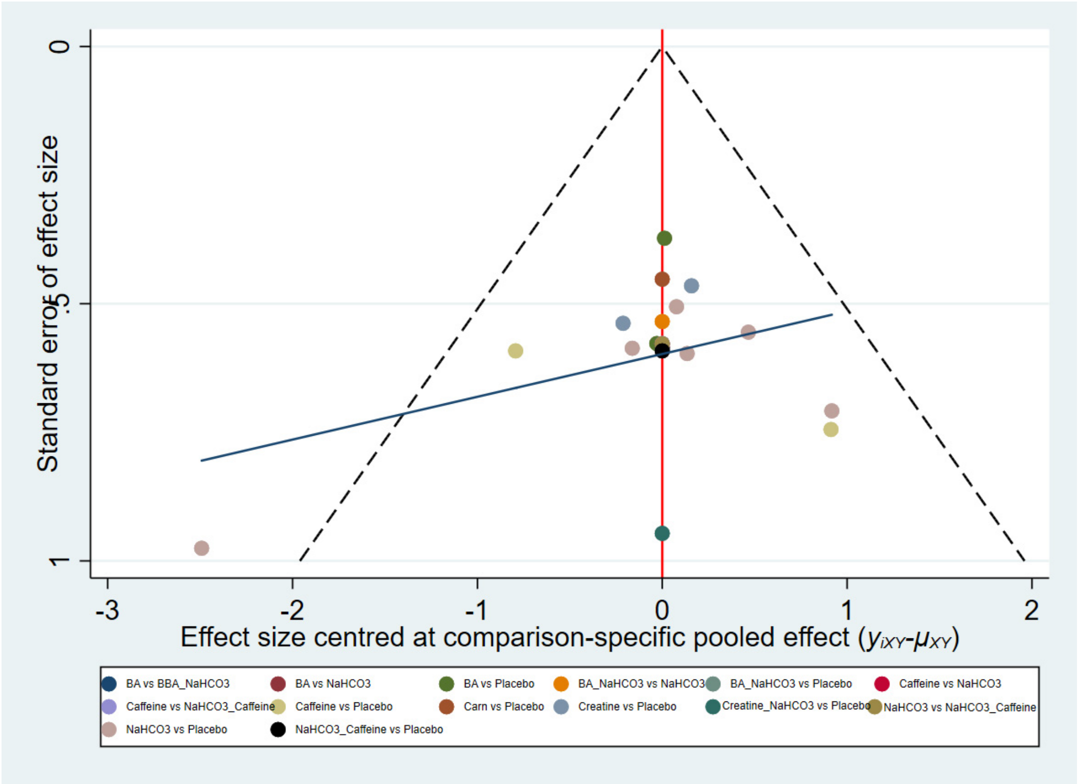

f

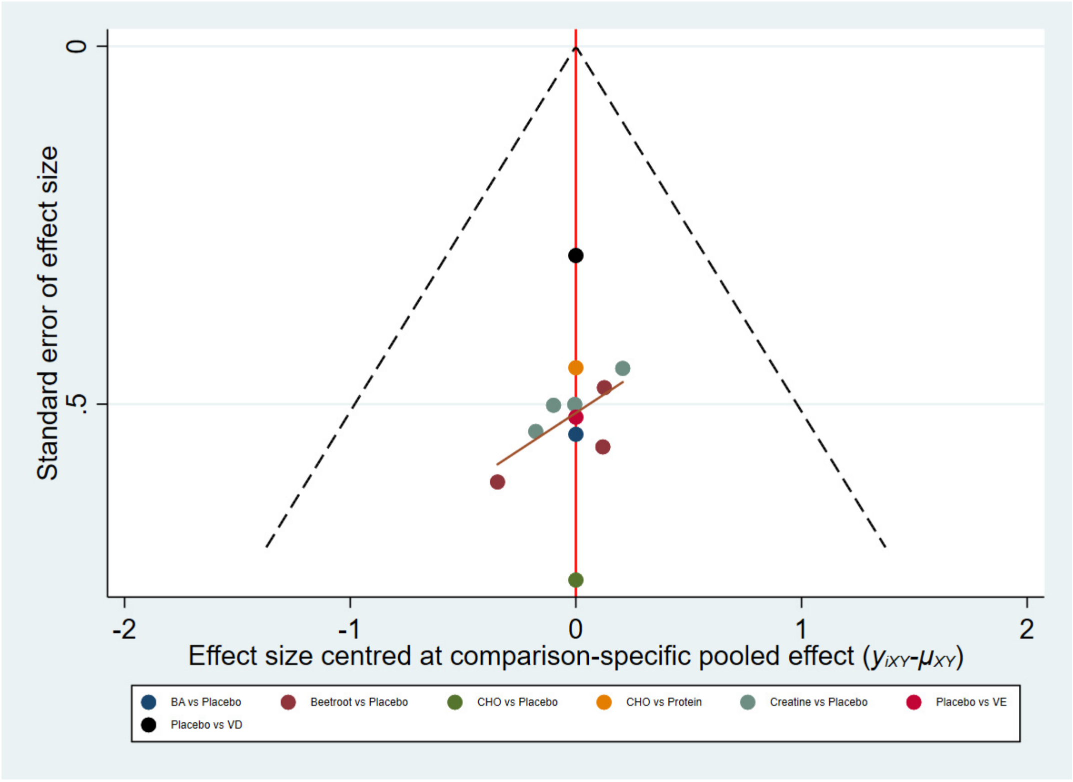

Figure S4. (continued)

g

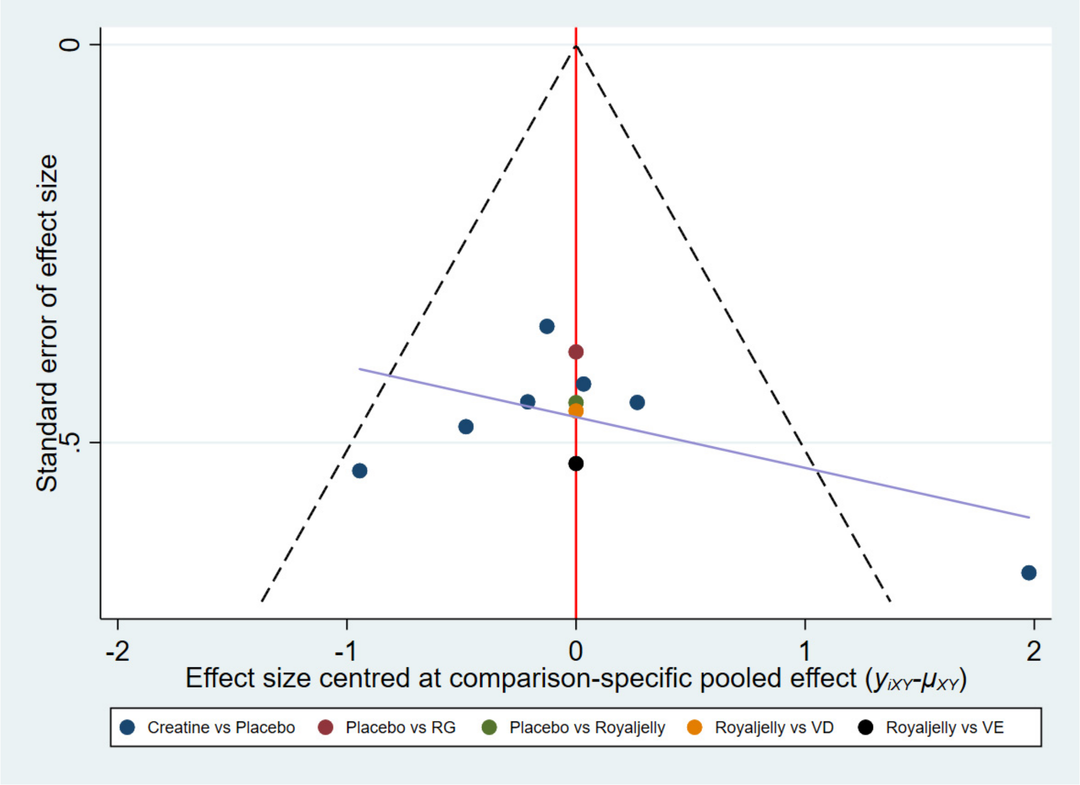

Note: BA: Beta-alanine, CHO: Carbohydrate, $NaHCO_3$: Sodium bicarbonate, Carn: L-Carnitine, VE: Vitamin E, RG: Rice germ, CSC: Chronic sodium citrate, VD: Vitamin D_3, Arg: L-arginine, Cit: L-Citrulline, BCAAs: Branched-Chain Amino Acids, CoQ10: Coenzyme Q10

Table S5. Global inconsistency and heterogeneity of each outcome

Outcomes	Consistency model					Inconsistency model					dDIC
	Dbar	Data points	pD	DIC	I^2	Dbar	Data points	pD	DIC	I^2	
50 m time	20.56	17	13.34	33.90	22%	20.60	17	13.38	33.98	22%	0.08
100 m time	34.87	27	13.17	48.04	25%	35.10	27	19.01	54.11	26%	6.07
200 m time	15.44	19	12.51	27.95	0%	17.91	19	16.36	34.27	0%	6.32
Blood lactate	44.55	43	21.82	66.37	6%	45.20	43	28.48	73.68	7%	7.31
Blood pH	21.71	22	10.07	31.78	3%	24.42	22	17.17	41.58	14%	9.80
Heart rate	9.01	13	7.94	16.95	0%	9.06	13	7.99	17.05	0%	0.10
Body mass	13.14	11	9.26	22.40	24%	13.21	11	9.25	22.45	24%	0.05

Table S6. Node-splitting results

Study	Mean Difference (95% CrI)			P-value
	Direct	Indirect	Network	
50 m time				
NA	NA	NA	NA	NA
100 m time				
Cit vs Arg	0.27 (-1.2,1.7)	0.25 (-1.9,2.4)	0.26 (-0.90,1.4)	0.99
Placebo vs Arg	1.7 (0.12,3.2)	1.7 (-0.41,3.7)	1.7 (0.45,2.9)	0.99
BA_NaHCO3 vs BA	-0.018 (-0.85,0.83)	0.16 (-0.84,1.2)	0.062 (-0.56,0.66)	0.78
NaHCO3 vs BA	0.17 (-0.95,1.3)	0.34 (-0.60,1.3)	0.27 (-0.42,0.98)	0.81
Placebo vs BA	0.24 (-0.43,0.94)	-0.040 (-1.1,0.99)	0.15 (-0.39,0.71)	0.64
NaHCO3 vs BA_NaHCO3	0.20 (-0.92,1.3)	0.21 (-0.83,1.3)	0.22 (-0.51,0.95)	0.98
Placebo vs BA_NaHCO3	-0.035 (-1.1,1.1)	0.17 (-0.72,1.1)	0.094 (-0.56,0.78)	0.77
Placebo vs Cit	1.4 (-0.11,2.9)	1.4 (-0.69,3.5)	1.4 (0.21,2.6)	0.99
Placebo vs NaHCO3	-0.22 (-1.3,0.90)	-0.032 (-1.0,0.96)	-0.12 (-0.84,0.60)	0.79
200 m time				
NA	NA	NA	NA	NA
Blood lactate				
Cit vs Arg	-1.3 (-2.8,0.20)	-0.44 (-2.1,1.1)	-0.88 (-2.,0.18)	0.46
Placebo vs Arg	-0.51 (-1.3,0.22)	-1.3 (-3.4,0.72)	-0.60 (-1.3,0.091)	0.46
BA_NaHCO3 vs BA	0.52 (-0.77,1.8)	0.71 (-0.41,1.8)	0.62 (-0.19,1.4)	0.83
NaHCO3 vs BA	0.58(-0.73,1.9)	0.96 (0.14,1.8)	0.84 (0.17,1.5)	0.61
Placebo vs BA	0.37 (-0.42,1.2)	-0.021 (-1.1,1.0)	0.25 (-0.39,0.85)	0.55
NaHCO3 vs BA NaHCO3	0.060 (-1.2,1.3)	0.32 (-0.72,1.4)	0.22 (-0.56,1.0)	0.74
Placebo vs BA_NaHCO3	-0.33 (-1.6,0.96)	-0.42 (-1.4,0.54)	-0.38 (-1.2,0.36)	0.90
NaHCO3 vs Caffeine	0.71 (-0.58,2.)	-0.43 (-1.2,0.34)	-0.14 (-0.82,0.54)	0.13
NaHCO3_Caffeine vs Caffeine	1.2 (-0.19,2.5)	0.69 (-0.49,1.9)	0.89 (-0.0022,1.8)	0.58
Placebo vs Caffeine	-1.0 (-1.7, -0.38)	0.043 (-1.0,1.1)	-0.73 (-1.3, -0.15)	0.09
Placebo vs Cit	-0.057 (-1.4,1.3)	0.76 (-0.91,2.4)	0.28 (-0.78,1.3)	0.45
NaHCO3_Caffeine vs NaHCO3	0.47 (-0.81,1.8)	1.4 (0.32,2.5)	1.0 (0.16,1.9)	0.26
Placebo vs NaHCO3	-0.43 (-0.99, 0.11)	-0.94 (-1.8, -0.17)	-0.59 (-1.1, -0.16)	0.29
Placebo vs NaHCO3_Caffeine	-2 (-3.5, -0.55)	-1.4 (-2.5, -0.43)	-1.6 (-2.5, -0.79)	0.53
Blood pH				
Placebo vs Caffeine	-0.086 (-1.1,0.91)	-0.88 (-1.9,0.20)	-0.44 (-1.2,0.29)	0.29
NaHCO3_Caffeine vs NaHCO3	-0.12 (-1.4,1.2)	-0.37 (-1.4,0.68)	-0.27 (-1.1,0.54)	0.75
Placebo vs NaHCO3	-0.98 (-1.5, -0.44)	-0.84 (-1.7, -0.015)	-0.94 (-1.4, -0.49)	0.78
Placebo vs NaHCO3_Caffeine	-0.77 (-2.1,0.58)	-0.61 (-1.7,0.43)	-0.66 (-1.5,0.13)	0.85
Heart rate				
NA	NA	NA	NA	NA
Body mass				
NA	NA	NA	NA	NA

Note: P-value < 0.05 may show significant inconsistency. BA: Beta-alanine, CHO: Carbohydrate, $NaHCO_3$: Sodium bicarbonate, Carn: L-Carnitine, VE: Vitamin E, RG: Rice germ, CSC: Chronic sodium citrate, VD: Vitamin D_3, Arg: L-arginine, Cit: L-Citrulline, BCAAs: Branched-Chain Amino Acids, CoQ10: Coenzyme Q10
